# Tumor cell–derived IFN spatially reprograms osteopontin-enriched macrophage niches to promote PARP inhibitor resistance

**DOI:** 10.1172/JCI199035

**Published:** 2026-03-06

**Authors:** Dan Liu, Kangjia Tao, Cheng Xu, Wen Yang, Chujun Cai, Cui Feng, Kairong Xiong, Sisi Wu, Yaying Lin, Zikun Peng, Jianhua Chi, Wen Pan, Qing Zhong, Jiahao Liu, Xiong Li, Xingzhe Liu, Dongchen Zhou, Ding Ma, Guang-Nian Zhao, Yu Xia, Yong Fang, Qinglei Gao

**Affiliations:** 1 National Clinical Research Center for Obstetrics and Gynecology, Department of Gynecological Oncology,; 2 Cancer Biology Research Center (Key Laboratory of the Ministry of Education, Hubei Provincial Key Laboratory of Tumor Invasion and Metastasis), and; 3 Department of Radiology, Tongji Hospital, Tongji Medical College, Huazhong University of Science and Technology, Wuhan, China.; 4 Department of Obstetrics and Gynecology, Wuxi Medical College, Jiangnan University, Wuxi, China.; 5 Department of Gynecology and Obstetrics, Central Hospital of Wuhan, Tongji Medical College, Huazhong University of Science and Technology, Wuhan, China.

**Keywords:** Immunology, Oncology, Biomarkers, Cancer, Immunotherapy

## Abstract

Poly (ADP-ribose) polymerase inhibitors (PARPis) benefit homologous recombination-deficient (HRD) malignancies, yet resistance remains a major challenge. Leveraging specimens from a prospective neoadjuvant niraparib monotherapy trial in treatment-naive, high-grade serous ovarian cancer, we integrated PhenoCycler-Fusion spatial profiling, scRNA-Seq, and multiplex immunohistochemistry to identify 2 therapeutic-modulated cellular neighborhoods: an IFN^+^ tumor cell–enriched niche that expands in resistant lesions and a niche enriched in tumor-associated macrophage (TAM) that persists but acquires enhanced immunosuppressive features. Mechanistically, sustained tumor cell–derived IFN induced osteopontin (SPP1) expression in TAMs via STAT signaling, creating immunosuppressive niches enriched in Tregs and myofibroblastic cancer–associated fibroblasts with intensified cell-cell interactions. SPP1 directly suppressed T cell signaling and effector function. High baseline SPP1^+^ cells predicted lower response rate (30.0% vs. 76.2%; *P* = 0.021) and shorter progression-free survival (median 13.5 vs. 28.3 months; *P* = 0.0006). In HRD mouse models, SPP1 blockade restored PARPi sensitivity, reversed acquired resistance, and enhanced T cell cytotoxicity—effects abrogated in immunodeficient mice, confirming immune dependence. These data establish a spatial IFN-SPP1 axis whereby persistent tumor cell IFN reprograms TAMs to promote PARPi resistance, position SPP1 as a key therapeutic target and prognostic biomarker for this therapy, and underscore therapeutic potential of microenvironment-targeted strategies to overcome PARPi resistance.

## Abstract 2

Tumor cells spatially reprogram macrophage neighborhoods via interferon to resist therapy, showing osteopontin blockade restores sensitivity.

## Graphical abstract

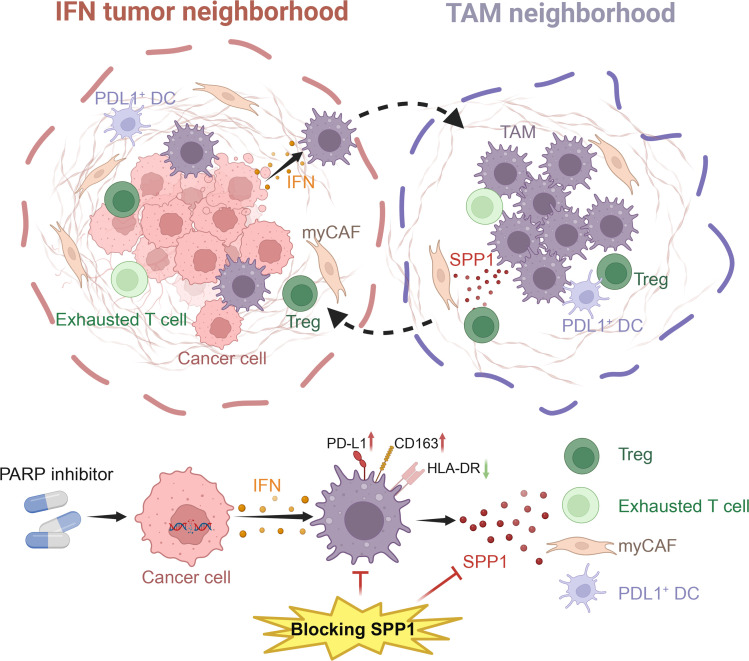

## Introduction

Immunotherapy has redefined the therapeutic landscape across multiple solid tumors, yet its clinical impact remains constrained by heterogeneous and often modest efficacy. Objective response rates to immune checkpoint inhibitors reach 30%–45% in melanoma and microsatellite-unstable colorectal cancer, whereas they fall below 25% in non–small-cell lung cancer and triple-negative breast cancer, and remain below 15% in ovarian cancer (1–3). In parallel, poly (ADP-ribose) polymerase inhibitors (PARPis) have become standard of care for homologous recombination-deficient (HRD) malignancies by inducing synthetic lethality, yet 40%–70% of patients ultimately develop primary or acquired resistance (4, 5). Although PARPis, in principle, can potentiate antitumor immunity through immunogenic cell death, early clinical efforts combining PARPis with anti–PD1/PD-L1 therapy have yielded only modest benefits, with objective response rates of 7.3%–21% in ovarian, non–small-cell lung and triple-negative breast cancers (6–9). This limitation points to immune-suppressive hubs within the tumor microenvironment (TME) that act beyond the PD-1/PD-L1 axis and curtail the synergistic potential of PARPi plus immunotherapy combinations.

Whereas an immunosuppressive TME is recognized as a driver of resistance to chemotherapy and immunotherapy, PARPi resistance has been attributed primarily to tumor-intrinsic mechanisms, including BRCA reversion, epigenetic restoration of homologous recombination proficiency, shieldin-complex loss, altered PARPi transport, or activation of prosurvival signaling (10). Emerging preclinical studies have begun to interrogate the influence of stromal and immune constituents, such as cancer-associated fibroblasts (CAFs), tumor-infiltrating T cells, and tumor-associated macrophages (TAMs), on PARPi sensitivity (6, 11, 12). Clinical investigation of the key players in human tissue, however, is hampered by limited posttreatment sampling and is confounded by the near-universal use of chemotherapy and antiangiogenic agents, which, themselves, profoundly remodel the microenvironment and obscure PARPi-specific effects (13). Moreover, the TME is not a collection of isolated cell types but a spatially choreographed ecosystem of cellular neighborhoods (CNs) whose collective interactions dictate therapeutic outcome (14, 15). Decoding the spatial architecture with cutting-edge technologies in longitudinal specimens exposed exclusively to PARPi and identifying the molecular hub that orchestrates resistance are, therefore, essential steps toward enhancing the efficacy of combined immune- and DNA damage–directed strategies.

This challenge is particularly acute in high-grade serous ovarian cancer (HGSOC), where HRD prevalence exceeds 50%, yet the disease remains the most lethal gynecologic malignancy despite widespread PARPi adoption (16, 17). Here, we leveraged specimens from the prospective Niraparib for the Neoadjuvant Treatment of Unresectable Ovarian Cancer (NANT) trial (ClinicalTrials.gov NCT04507841) to perform an integrated spatial and single-cell analysis of treatment-naive HGSOC before and after PARPi exposure, eliminating confounding chemotherapy effects. Through PhenoCycler-Fusion (PCF) spatial profiling, single-cell RNA-Seq (scRNA-Seq), and functional validation, we identify 2 dynamically interacting CNs that co-evolve during PARPi therapy: an IFN^+^ tumor cell–enriched niche that expands in resistant lesions, and a spatially adjacent TAM-enriched niche that persists but undergoes osteopontin-mediated (SPP1–mediated) reprogramming toward enhanced immunosuppression. Mechanistically, sustained tumor cell–derived IFN drives SPP1 expression in TAMs, creating an immunosuppressive axis that involves FOXP3^+^PD1^+^ effector Tregs and αSMA^+^ myofibroblastic CAFs (myCAFs) and that impairs T cell function and predicts poor clinical outcome. These findings establish spatially organized microenvironmental remodeling as a critical contributor to PARPi resistance and nominate SPP1 as a therapeutically actionable target to restore PARPi sensitivity.

## Results

### Samples from the prospective NANT trial.

**Figure 1:**
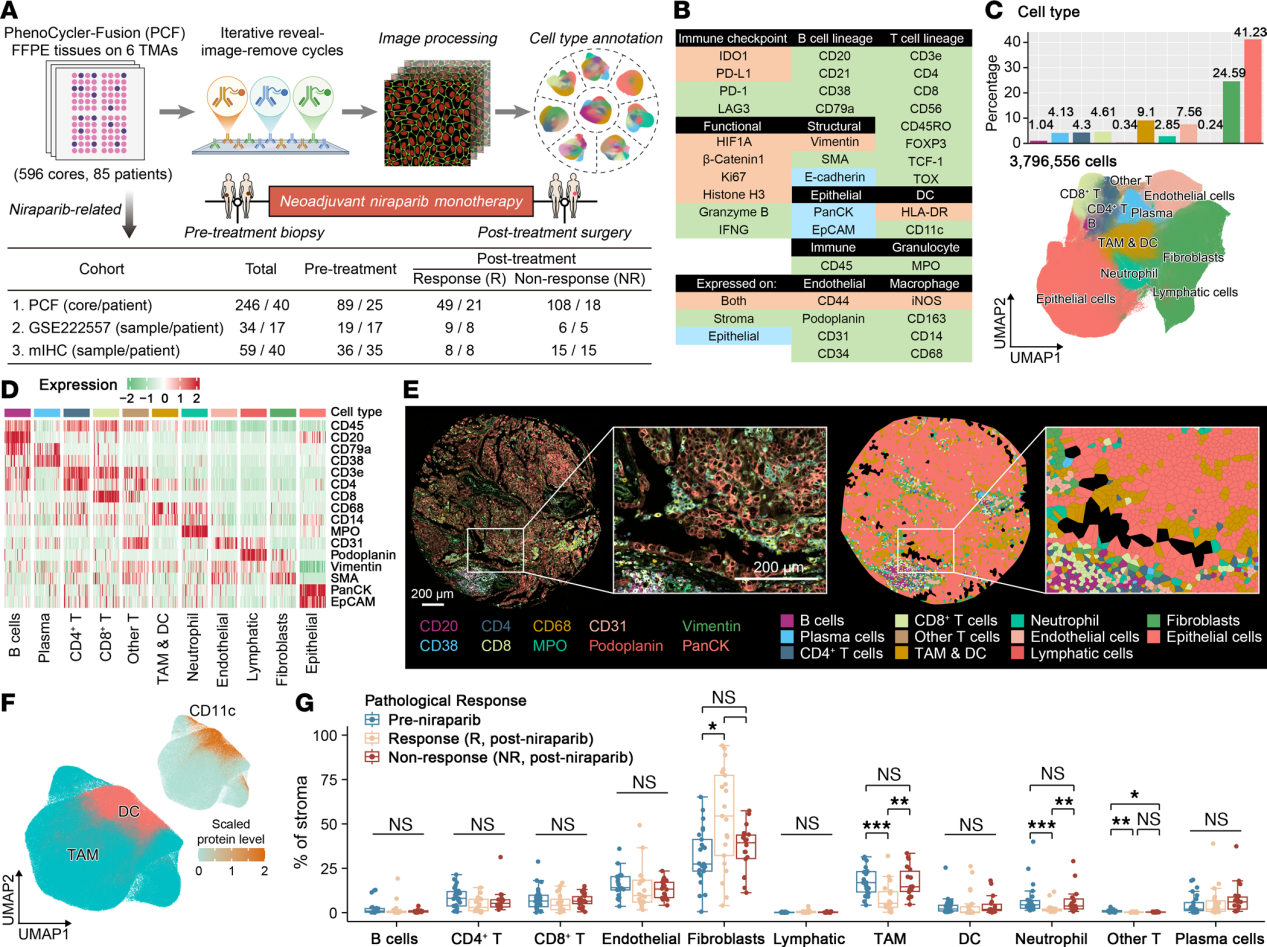
TAMs persist in niraparib nonresponse tumors. (**A**) Experimental workflow for longitudinal tumor sampling and multimodel profiling in the NANT trial. (**B**) Antibody panel design for 39-protein spatial phenotyping, with background colors marking epithelial (blue) or TME (green) cells, or both (orange). (**C**) Uniform manifold approximation and projection (UMAP) of single cells color-coded by major lineage (lower), with bar plots quantifying relative cellular abundances (upper). (**D**) Heatmap of *z* score–normalized protein levels for canonical lineage markers across cell clusters. (**E**) PCF images (left: multiplex marker overlay; right: Voronoi tessellation) from a representative region (TMA4_K_3). (**F**) UMAP of single cells color coded by TAM and DC lineages, with plots showing the normalized protein levels of CD11c. (**G**) Comparative analysis of cell-type proportions across treatment phases and response categories. Pretreatment (*n* = 25), post-niraparib responders (R) (*n* = 21), and nonresponders (NR) (*n* = 18) were analyzed. Box plots show medians with IQRs. Statistical significance was determined by 2-tailed Wilcoxon rank-sum test with Benjamini-Hochberg correction. *Padj < 0.05, **Padj < 0.01, ***Padj < 0.001.

To dissect the microenvironmental basis of differential responses of PARP inhibition, we used PCF spatial protein profiling, scRNA-Seq, and multiplex immunohistochemistry (mIHC) analysis leveraging samples from our prospective NANT trial (18, 19) (Figure 1A). In this phase II, single-arm clinical trial, HRD testing was used to screen 127 patients with HGSOC who were assessed by CT or laparoscopic exploration as unlikely to achieve optimal cytoreduction. Among these, patients with HRD-positive status received two 28-day cycles of niraparib monotherapy, with a median 11 days interval from final dose to the following interval debulking surgery. We collected FFPE specimens from 85 patients, which were prepared into 6 tissue microarrays comprising 596 tissue cores. For pretreatment analyses, patient-level Response Evaluation Criteria in Solid Tumors (RECIST), version 1.1 (RECIST 1.1) response criteria were applied; for posttreatment analyses, we used lesion-level histopathologic response classification (see Methods) (20, 21). After excluding patients who did not receive complete niraparib treatment and omitting normal contralateral tissues, the PCF analysis focused on 246 pre- and posttreatment cores from 40 patients (Figure 1A), using a 39-marker antibody panel (Figure 1B). Technical validation confirmed robust detection of 38 protein targets, with CD56—a canonical NK cell marker—excluded due to assay failure (Supplemental Figure 1A; supplemental material available online with this article; https://doi.org/10.1172/JCI199035DS1).

### TAMs persist in niraparib nonresponse tumors.

After cell segmentation, quality control, intensity normalization, and data integration across 6 tissue microarrays, 3,796,556 cells were identified (Supplemental Figure 1B). Major cell populations—including epithelial cells, fibroblasts, CD4^+^, CD8^+^ T cells, and myeloid subsets—were annotated via canonical markers (Figure 1, C and D), with spatial distributions visualized through immunofluorescence and Voronoi tessellation (22) (Figure 1E). TAMs and DCs were further distinguished on the basis of CD11c protein levels (Figure 1F).

For each sample, 1–4 tissue cores were procured, and cellular proportions were averaged across cores to yield a single lesion-level datum for downstream analyses. Hierarchical clustering of cellular proportions revealed no associations with age, stage, sampling site, or BRCA mutation status, but exhibited significant variability across treatment phases and lesion-level pathologic response categories (Supplemental Figure 1C). Specifically, the baseline TME composition showed no difference between clinical responders and nonresponders (Supplemental Figure 1D), whereas niraparib treatment induced substantial adaptive alterations (Figure 1G and Supplemental Figure 1E). Compared with pretreatment baselines, posttreatment responsive lesions had the most significant decrease in TAM abundance, whereas posttreatment nonresponsive lesions had similar levels of TAM infiltration (Figure 1G). Although niraparib is 1 of the more myelosuppressive PARPis, posttreatment TAM levels showed no association with bone marrow status as assessed by patients’ hematological tests (Supplemental Figure 1F). These findings indicate that effective niraparib therapy is associated with a reduced TAM prevalence, and persistent TAM enrichment is associated with therapeutic resistance.

### IFN-responding macrophages are enriched in resistant lesions.

**Figure 2:**
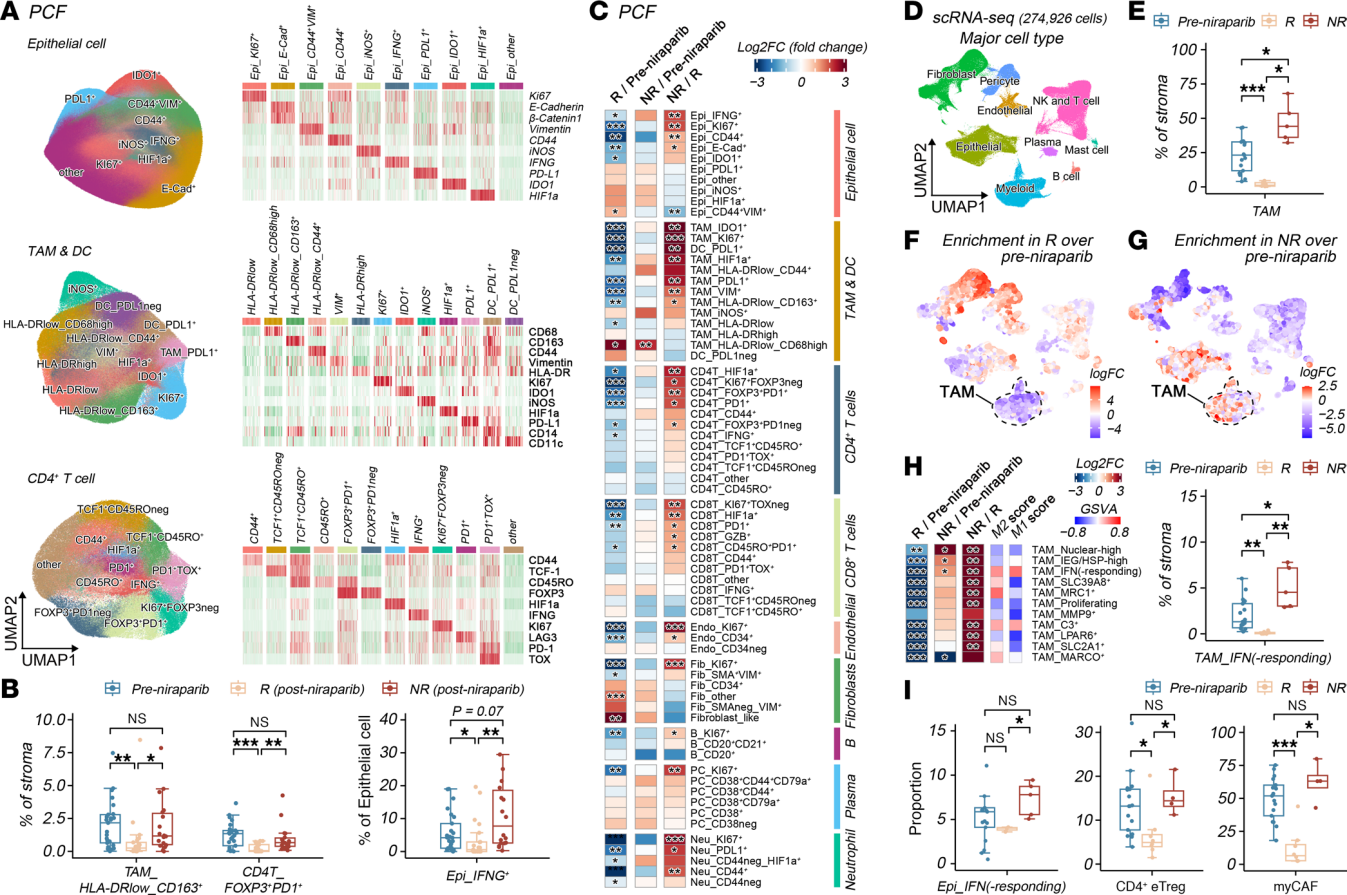
IFN-responding macrophages are enriched in resistant lesions. (**A**) Integrated Uniform manifold approximation and projection (UMAP) visualization with heatmap showing color-coded cell subtype annotation of epithelial (epi) cells, TAMs and DCs, and CD4^+^ T cells derived from PCF analysis; right-side panel displays *z*-scored marker expression confirming lineage-specific signatures. (**B**) Quantitative box plots representing the quantified percentages of specific cell subsets in pretreatment (*n* = 25), post-niraparib responder (R) (*n* = 21) and post-niraparib nonresponder (NR) (*n* = 18) clinical specimens. (**C**) Heatmaps illustrating the log2-fold changes (Log2FC) in cell abundance between post-niraparib R (*n* = 21) versus pretreatment (*n* = 25) (left), post-niraparib NR (*n* = 18) versus pretreatment (*n* = 25) (middle), and post-niraparib NR (*n* = 18) versus R (*n* = 21) (right) across all cell subtypes. (**D**) UMAP projection of scRNA-Seq data annotated into major cell types. (**E**) Proportion quantification of TAM in pretreatment (*n* = 17), post-niraparib R (*n* = 8), and post-niraparib NR (*n* = 5) clinical specimens. (**F** and **G**) UMAP plots of major cell types with Milo differential abundance testing results overlaid in color gradient, depicting log fold changes of post-niraparib R (**F**) or NR (**G**) relative to pretreatment (Pre) samples. (**H**) Heatmap illustrating the log_2_ fold changes in cell abundance of specific TAM subclusters across 3 comparisons (R versus Pre; NR versus Pre; NR versus R). Adjacent columns display the gene set variation analysis (GSVA) scores for M1 and M2 polarization signatures across identified TAM subtypes. Box plot shows quantification of the stromal proportion of the IFN-responding TAM cluster across pretreatment (*n* = 17), post-niraparib R (*n* = 8), and post-niraparib NR (*n* = 5) clinical specimens. (**I**) Box plot quantifying the proportion of the indicated cell subsets across pretreatment (*n* = 17), post-niraparib R (*n* = 8), and post-niraparib NR (*n* = 5) clinical specimens. Wilcoxon rank-sum test. Statistical significance was determined by 2-tailed Wilcoxon rank-sum test, and the *P*adj values were calculated by Benjamini-Hochberg correction (**B**, **C**, **E**, and **H**). *Padj < 0.05, **Padj < 0.01, ***Padj < 0.001.

Cellular populations within the TME exhibit substantial phenotypic and functional heterogeneity. To dissect this complexity, we performed unsupervised subclustering on each major cell type (Figure 2A and Supplemental Figure 2). Within epithelial cells (primarily tumor cells), we identified subsets characterized by distinct expression profiles of IFNG, PDL1, KI67, E-cadherin, and other markers, likely representing distinct functional states. Within macrophages, subsets such as PDL1^+^ TAMs, IDO1^+^ TAMs, and VIM^+^ TAMs were identified, which have been implicated in immunosuppressive functions (22). Notably, M2-like macrophages typically exhibited low HLA-DR expression, a feature observed in classical M2-like CD163^+^ TAM. Among CD4^+^ T cells, FOXP3^+^ Tregs were further stratified into PD1^+^ effector Tregs (eTregs), a subset recognized for potent immunosuppressive activity (18). Tumor-associated fibroblasts included αSMA^+^VIM^+^ myofibroblasts (i.e., myCAFs), which have been reported to drive immune exclusion via TGF-β pathway activation, extracellular matrix remodeling, and aberrant angiogenesis (23). Endothelial cells were categorized into CD34^+^ precursor/smaller and CD34^−^ mature blood vessels (24).

Comparative analysis of cellular abundance indicated that numerous cell subtypes were decreased in posttreatment responsive lesions compared with pretreatment baselines, including CD44^+^, E-cadherin^+^, and IDO^+^ tumor cell subsets, multiple immunosuppressive TAM populations, eTregs, PD1^+^ CD8 T cells, myCAFs, CD34^+^ endothelial cells, PDL1^+^ neutrophils, and various KI67^+^ proliferating cells. In contrast, the levels of these cells remained largely unchanged in posttreatment resistant residual lesions compared with pretreatment baselines (Figure 2, B and C). Although the persistence of multiple cell types in resistant lesions, such as TAMs, eTregs, and myCAFs, may still contribute to therapeutic resistance, this likely represents a concomitant effect of residual tumor burden. Notably, IFNG^+^ tumor cells were significantly decreased in posttreatment responsive lesions but showed an upward trend in residual lesions (*P* = 0.07) compared with pretreatment baselines, suggesting that resistant tumors may preferentially enrich for IFNG^+^ tumor cells to a certain extent (Figure 2, B and C).

To further delineate microenvironmental determinants of therapeutic response, pre- and post-niraparib specimens from our NANT scRNA-Seq dataset (GSE222557) were reanalyzed. The scRNA-Seq data contained transcriptomic profiles for 274,926 cells, with a median depth of 11,561 reads and 2,792 genes per cell (Supplemental Figure 3, A and B). Annotation using canonical markers resolved major cell types (Figure 2D and Supplemental Figure 3C). Comparative analysis of cellular composition ratios revealed selective TAM depletion in posttreatment responsive tumors, and TAMs accumulated in nonresponsive tumors compared with pretreatment samples, with minimal changes in other major cell types (Figure 2E and Supplemental Figure 3, D and E). Graph-based differential abundance analysis demonstrated that although macrophages were decreased at the global level in posttreatment responsive lesions (Figure 2F), marked differences at the cellular subtype level existed in posttreatment resistant lesions (Figure 2G). We therefore performed subcluster annotation across all major cell lineages in the scRNA-Seq data (Supplemental Figure 4, A and B). MyCAFs were recognized for the expression of ACTA2 (α-SMA), and CD4^+^ eTregs were distinguished from resting Tregs by their high expression of *TNFRSF9*, *TNFRSF18*, *IL2RA,* and *PD1* (25), aligning with our classifications at the protein level (via PCF) (Supplemental Figure 4, A and B). IFN-responding TAMs, which expressed IFN-inducible genes such as *ISG15*, *MX1*, and *IFIT1*, exhibited a canonical M1-like phenotype yet concurrently displayed elevated M2-like characteristics, reflecting their complex functional state (Figure 2H). Systematic comparative analysis of cellular subtype proportions further showed that IFN-responding, immediate early gene/heat shock protein-high (IEG/HSP)-high, and nuclear-high TAMs were the only 3 populations significantly enriched in posttreatment nonresponsive tumors compared with pretreatment samples, whereas other cell subtypes, such as IFN-responding epithelial cells, CD4^+^ eTregs, and myCAFs, were maintained at similar levels in resistant lesions (Figure 2, H and I, and Supplemental Figure 4C). Complementary single-cell mIHC using alternative markers for eTregs (CD4^+^FOXP3^+^TNFRSF9^+^) (25) and myCAF (FAP^+^ACTA2^+^) (26) further validated these findings (Supplemental Figure 4D). Collectively, the multimodal data indicate that IFNG-expressing tumor cells show an enrichment trend in posttreatment resistant lesions, IFN-responding TAMs are significantly increased, and various immunosuppressive cell types, including eTregs and myCAFs, persist in resistant lesions, together constituting a resistant microenvironment.

### IFNG^+^ tumor cell–enriched and TAM-enriched CNs are associated with niraparib resistance.

**Figure 3:**
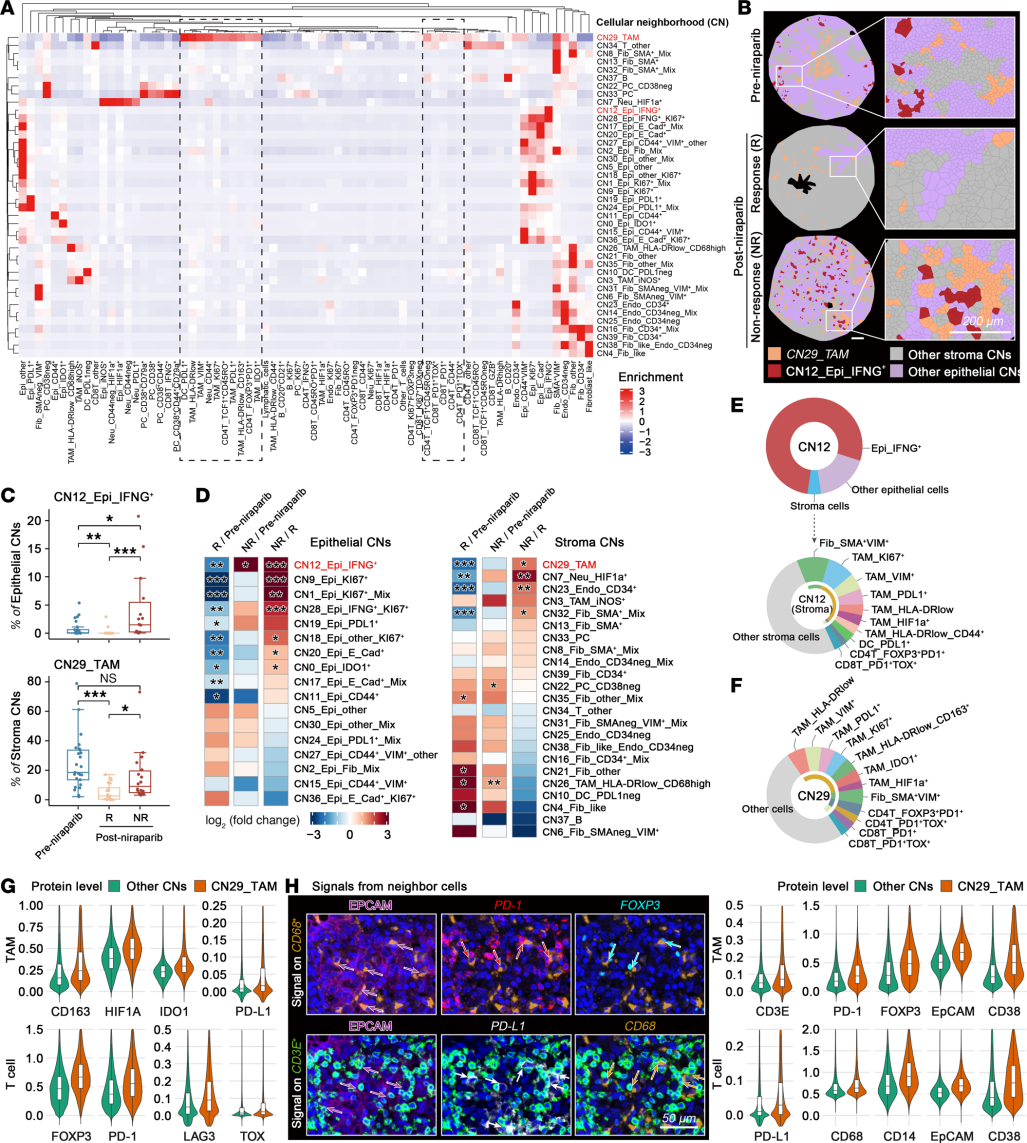
IFNG^+^ tumor cell–enriched CNs and TAM-CNs are associated with niraparib resistance. (**A**) Forty CNs were computationally identified through k-nearest neighbor graph clustering of 71 annotated cell subtypes. CN annotations reflect dominant cellular compositions. (**B**) Therapeutic modulation of CN29_TAM and CN12_Epi_IFNG^+^. Voronoi diagrams illustrating spatial distributions of CN29 and CN12. (**C**) Box plots quantifying proportions of CN12_Epi_IFNG^+^ within epithelial (Epi) CNs and CN29_TAM within stroma CNs in pretreatment (Pre) (*n* = 25), post-niraparib responder (R) (*n* = 20), and post-niraparib nonresponder (NR) (*n* = 18) clinical specimens. (**D**) Heatmaps illustrating the log2-fold changes in abundance of epithelial CNs (left) or stroma CNs (right) across 3 comparisons (R versus Pre; NR versus Pre; NR versus R). (**E** and **F**) Donut plots displaying cellular composition within CN12_Epi_IFNG^+^ (**E**) and CN29_TAM (**F**). (**G**) Violin plots comparing the normalized protein expression profiles of TAMs and T cells within CN29 versus other CNs. (**H**) Multiplex immunofluorescence co-localization analyses. Upper panel: Spatial interactions of CD68^+^ cells (TAMs, orange) with adjacent EPCAM^+^, PD-1^+^, or FOXP3^+^ cells. Lower panel: CD3E^+^ (T cells, green) with adjacent EPCAM^+^, PD-L1^+^, or CD68^+^ cells. The violin plots compare normalized protein signals detected on TAMs (upper) and T cells (lower) within CN29 versus other CNs. Statistical significance was determined by 2-tailed Wilcoxon rank-sum test with Benjamini-Hochberg correction (**C** and **D**); *P*adj values are reported. *Padj < 0.05, **Padj < 0.01, ***Padj < 0.001.

The coordinated variation in cell types suggests an organized immunosuppressive interplay within the therapy-refractory TME. To delineate these spatially structured functional ecosystems, we applied CN analysis, an advanced spatial profiling technique (Supplemental Figure 5A) (27). With optimal cluster numbers of CNs determined by the elbow method (Supplemental Figure 5B), we identified 40 distinct CNs (Figure 3A). The baseline abundance of CNs in treatment-naive samples did not differ between niraparib responder (partial response [PR]) and nonresponders (stable disease [SD] and progressive disease [PD])tumors, as assessed by RECIST 1.1 (Supplemental Figure 5, C and D). Critically, CN12, dominated by IFNG^+^ tumor cells, was the only CN that significantly increased in nonresponsive tumors and decreased in responsive lesions compared with pretreatment baselines, based on lesion-level pathologic response categories (Figure 3, B−D). CN12 comprised, on average, 77.73% IFNG^+^ tumor cells, 17.74% other tumor cells, and 4.53% stromal cells (Figure 3E, upper). Among the stromal cell components within CN12, approximately half consisted of multiple immunosuppressive TAM subsets (HLA-DR^low^, VIM^+^, PDL1^+^, KI67^+^, HIF1α^+^), α-SMA^+^VIM^+^ myCAFs, FOXP3^+^PD1^+^ eTregs, PDL1^+^ DCs, and PD1^+^TOX^+^ exhausted CD8^+^ T cells, indicating that IFNG-expressing tumor cells engage in intimate interactions with these stromal cells to constitute a functional unit (Figure 3E, lower). Interestingly, these same stromal cell populations constituted the primary components of CN29 (TAM-enriched neighborhoods [TAM-CNs]) (Figure 3F), suggesting potential interactions between CN12_Epi_IFNG^+^ and CN29_TAM.

Among stromal CNs, CN29_TAM exhibited the highest abundance across ovarian cancer samples (Supplemental Figure 5E). CN29_TAM markedly decreased in post-niraparib responsive tumors, whereas it was sustained in nonresponsive lesions relative to pretreatment baselines (Figure 3, B−D). Multiple immunosuppressive TAM subsets along with myCAFs and eTregs were uniquely enriched in CN29 (Figure 3A), indicating a functional synergy in immune suppression. Quantitative analysis showed elevated CD163, HIF1α, IDO1, and PD-L1 protein levels in CN29-localized TAMs, whereas T cells in CN29 displayed upregulated FOXP3, PD-1, LAG3, and TOX; both findings are consistent with an immunosuppressive environment (Figure 3G).

To investigate direct cell-cell interactions, we examined protein co-localization, where protein signals from adjacent cells appear on interacting partners, indicating cellular communication (28). The candidate interactions were carefully reviewed against the original multichannel images to exclude spurious overlaps arising, for example, from incomplete cell segmentation or residual autofluorescence. This analysis revealed that TAMs in CN29 demonstrated enhanced direct interactions with CD3E^+^, PD-1^+^, FOXP3^+^, EPCAM^+^, and CD38^+^ cells. Correspondingly, T cells in CN29 exhibited heightened interaction with PD-L1^+^, CD68^+^, CD14^+^, EPCAM^+^, and CD38^+^ cells (Figure 3H). These results define CN29 as an immunosuppressive niche with intense intercellular communication. Collectively, these results highlight the differential adaptive responses to niraparib, whereby the posttreatment increase of IFNG^+^ tumor cell-centric ecosystems and the persistent presence of associated TAM-centric cellular ecosystems may contribute to niraparib resistance.

### Enhanced spatial interaction between CNs in niraparib-resistant lesions.

**Figure 4:**
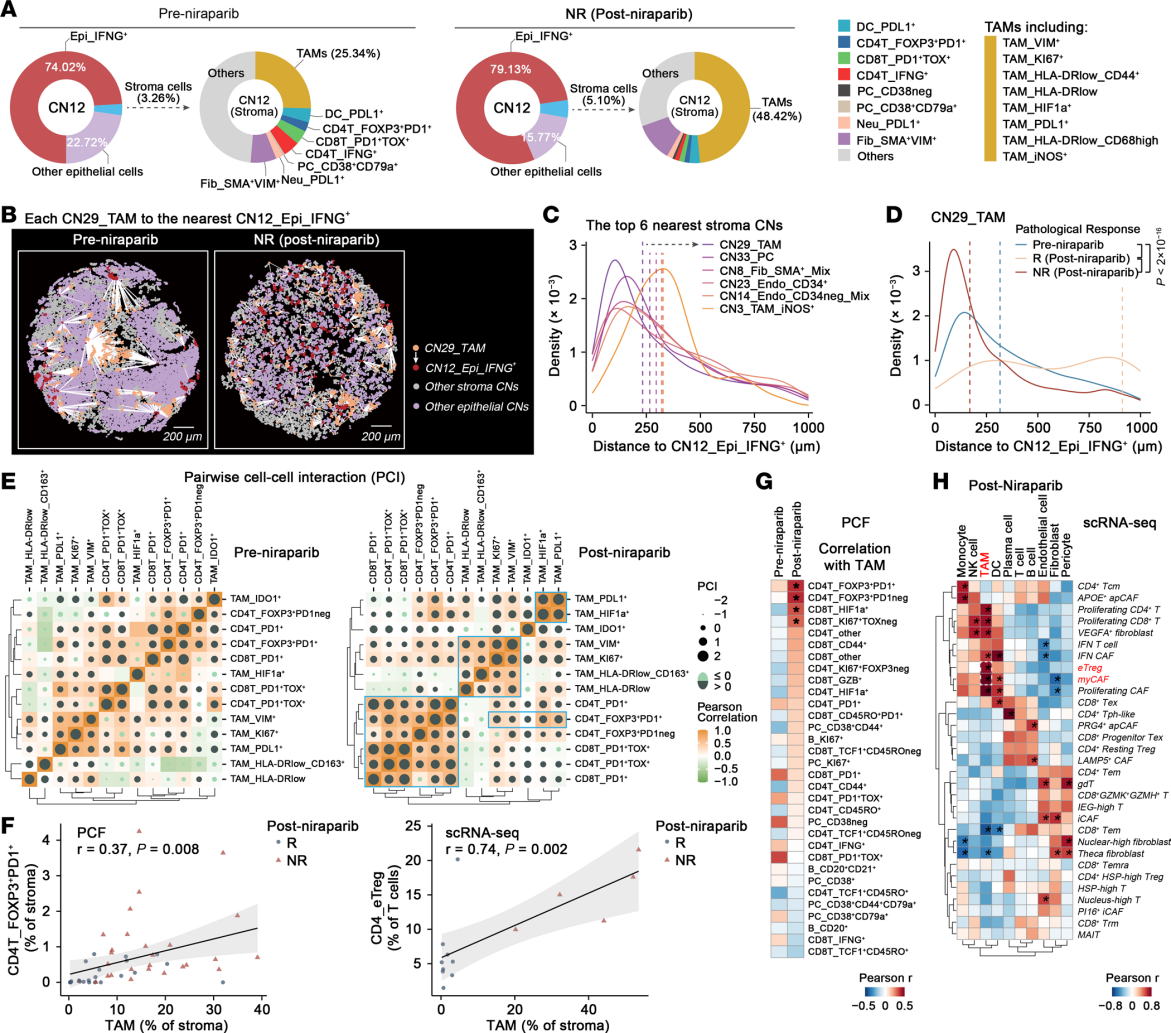
Enhanced spatial interaction between CNs in niraparib-resistant lesions. (**A**) Donut plots illustrating the cellular constituents of CN12_Epi_IFNG^+^ in pretreatment (left) and post-niraparib nonresponder (NR) (right) specimens. (**B**) Representative nearest-neighbor distances images of the proximity of CN29_TAM to CN12_Epi_IFNG^+^ with color-coded phenotypes in pretreatment (left) and post-niraparib NR (right) specimens. (**C**) Density plots illustrating the distribution of distances from all stromal CN centroids to their nearest-neighbor CN12_Epi_IFNG^+^ aggregated across all samples. The top 6 nearest stromal CNs are shown, with dotted vertical lines representing the median distance for each respective CN. (**D**) Density plots comparing the distance from the CN29_TAM to CN12_Epi_IFNG^+^ across clinical cohorts: pretreatment (blue), responders (R) (light orange), and nonresponders (dark red). Statistical significance was determined by Kolmogorov-Smirnov test. (**E**) Heatmaps of Pearson correlation coefficients (color of the square) and pairwise cell-cell interactions scores (circle size) among CN29-TAM niche components. Left: Pretreatment samples (*n* = 25). Right: Post-niraparib samples (*n* = 39). (**F**) Scatter plots illustrating the correlation between total TAM frequency (of stroma) and the proportion of CD4^+^FOXP3^+^PD1^+^ T cell subsets following niraparib treatment based on PCF (*n* = 49; left) and scRNA-Seq (*n* = 14; right), include both R and NR lesions. (**G**) Covariation of TAMs and immune cell abundances across pretreatment (*n* = 25; left) and post-niraparib (*n* = 49; right) samples, based on PCF data. Pearson correlation analysis. *P < 0.05. (**H**) Covariation of cell abundances within niraparib posttreatment tumors (*n* = 14), based on scRNA-Seq. Pearson correlation analysis was used. *P < 0.05.

To investigate the spatial architectural differences in CN12_Epi_IFNG^+^ and CN29_TAM before and after niraparib treatment, we first analyzed compositional variations within CN12. The results revealed that in posttreatment resistant lesions, the proportions of both IFNG^+^ tumor cells and stromal cells within CN12 increased modestly compared with pretreatment baselines. Notably, within the stromal compartment, the TAM fraction rose from 25.34% to 48.42%, suggesting that cellular interactions between IFNG^+^ tumor cells and TAMs were enhanced within the CN12_Epi_IFNG^+^ neighborhood in resistant lesions (Figure 4A). Conversely, in the CN12 of responsive lesions, the proportion of IFNG^+^ tumor cells decreased, whereas stromal components were completely devoid of TAMs, eTregs, and other immunosuppressive elements (Supplemental Figure 6A). Nearest-neighbor analysis demonstrated that CN29_TAM was the closest stromal neighborhood to CN12_Epi_IFNG^+^ (Figure 4, B and C). Moreover, the distance distribution between CN29_TAM and CN12_Epi_IFNG^+^ became significantly shorter in posttreatment resistant lesions, whereas it increased in responsive lesions (Figure 4D). These results demonstrate a robust spatial interaction between CN29_TAM and CN12_Epi_IFNG^+^, an interaction that is eliminated in responsive lesions but intensified in resistant lesions.

The coordinated dynamics and spatial relationship among TAMs, eTregs, and myCAFs motivated further investigation into their interplay during the development of niraparib resistance. To this end, pairwise cell-cell interactions were quantified, with positive values indicative of enhanced intercellular communication (Supplemental Figure 6B) (22). Focusing on cell types within CN29_TAM neighborhoods, analysis revealed that interactions among Tregs, PD1^+^ T cells, PD1^+^TOX^+^-exhausted T cells, and various TAM subsets were strengthened in post-niraparib tumors compared with treatment-naive samples (Figure 4E). A notable finding was the significant positive correlation between TAM abundance and Treg infiltration across all posttreatment tumors (Figure 4F), a pattern not observed in untreated tumors (Figure 4, F and G). Given that both cell types were depleted in responsive lesions but persisted in resistant lesions, this posttreatment co-occurrence pattern suggests a coordinated remodeling process. Validation via scRNA-Seq confirmed covariation among eTregs, myCAFs, and TAMs in niraparib-treated tumors (Figure 4, F and H). This finding was supported by mIHC analysis of matched fields of view in serial sections (Supplemental Figure 6C).

The baseline association of this triad in treatment-naive HGSOC was further validated in larger cohort of 130 tumors, using the GSE180661 dataset (29) (Supplemental Figure 6D), highlighting its role as a conserved immunosuppressive interplay. Collectively, these results demonstrate that in niraparib-resistant lesions, intercellular interactions are enhanced both between and within the CN12_Epi_IFNG^+^ and CN29_TAM CNs.

### The IFN-SPP1 axis orchestrates TME reprogramming in PARPi-resistant tumors.

Alterations in cellular composition and spatial interactions suggest functional reprogramming of cells following treatment. Consistent with this hypothesis, the intratumoral heterogeneity of TAM subtypes, as calculated by the diversity of their signaling pathways, decreased after niraparib treatment in both responders and nonresponders, suggesting a more homogeneous state of TAMs after niraparib treatment (Supplemental Figure 7A). However, divergent pathway activation was observed between these 2 groups, leading to increased intertumoral heterogeneity. Specifically, multiple functional pathways within TAMs were upregulated in responsive tumors, whereas these pathways were suppressed in nonresponsive lesions (Supplemental Figure 7B). Pathways associated with TNF-α signaling via NF-κB, lymphocyte activation, and leukocyte cell-cell adhesion exhibited this dichotomy (Supplemental Figure 7C). These data suggested that TAMs in nonresponsive lesions acquire enhanced immunosuppressive functions after niraparib treatment.

**Figure 5:**
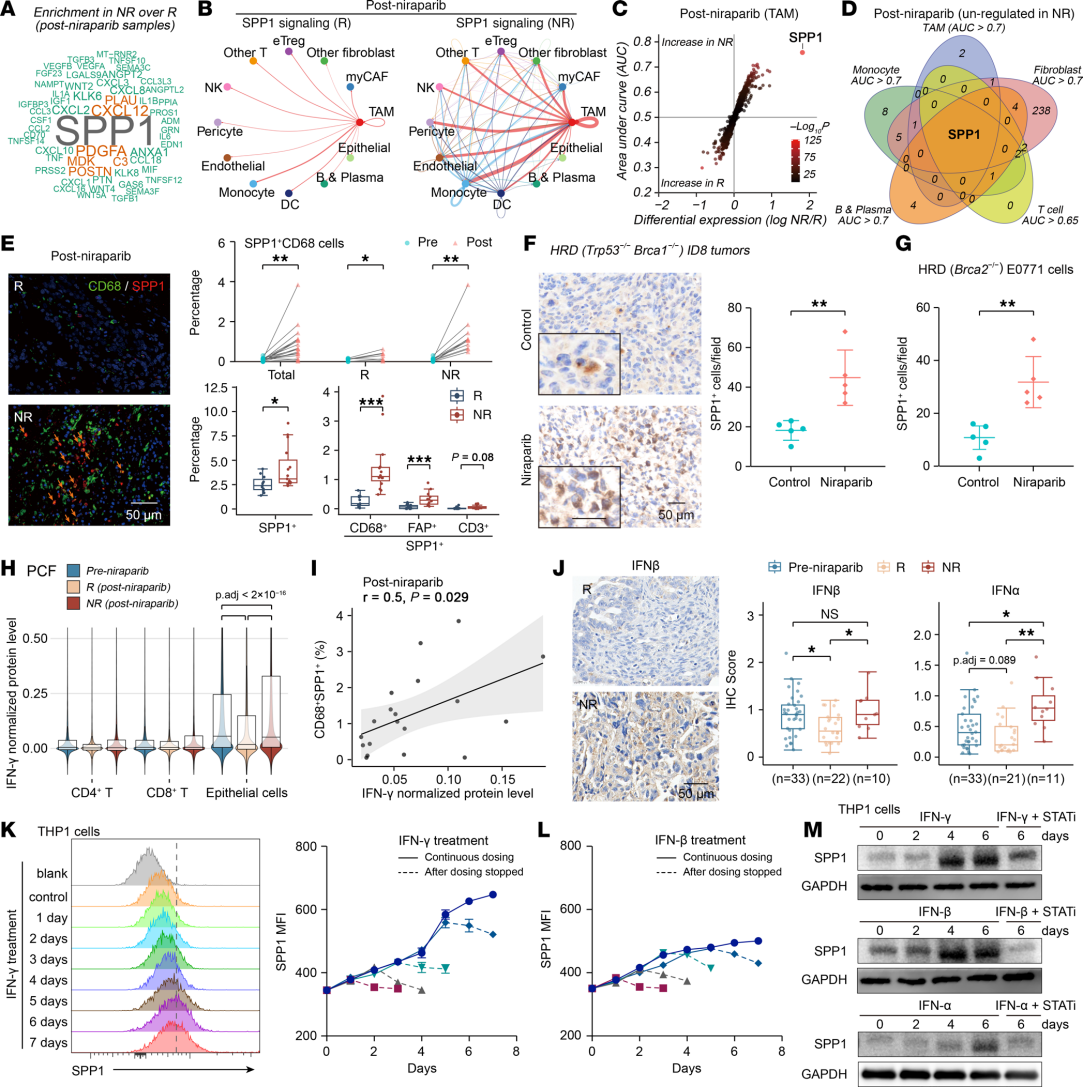
IFN-SPP1 axis orchestrates TME reprogramming in PARPi-resistant tumor. (**A** and **B**) CellChat analysis of secreted ligand-receptor interactions. Word clouds depict enriched signaling pathways (**A**) and circle plots visualize the SPP1 network (**B**) in nonresponder (NR) (*n* = 5) compared with responder (R) (*n* = 9) within niraparib posttreatment tumors. B, blood. (**C** and **D**) Differentially expressed genes in TAMs (**C**), and Venn diagram illustrating the top upregulated genes across various cell types (**D**) in NR (*n* = 5) compared with R (*n* = 9) within niraparib posttreatment tumors. (**E**) Representative immunofluorescent images with arrows indicating CD68^+^SPP1^+^ cells (left). Quantification of indicated cell proportions between pre- and post-niraparib tumors in total (*n* = 19 pairs), response (*n* = 8 pairs) and nonresponse group (*n* = 11 pairs) using a paired *t* test (upper right), and between R and NR in posttreatment tumors (*n* = 26−27) using Wilcoxon rank-sum test (lower right). (**F** and **G**) IHC quantification of SPP1^+^ cells per field (×40 magnification; *n* = 5 mice/group) in niraparib treated *Trp53^−/−^Brca1^−/−^* ID8 (**F**) and *Brca2^−/−^* E0771 (**G**) mouse models. Statistical significance was determined by Wilcoxon rank-sum test. (**H**) Violin plots show PCF-derived, normalized IFN-γ protein level across indicated cells, compared among pretreatment (*n* = 25), post-niraparib R (*n* = 20), and post-niraparib NR (*n* = 18). (**I**) Correlation between CD68^+^SPP1^+^ cell proportion and normalized IFN-γ protein level in post-niraparib tumors (*n* = 19). Pearson correlation analysis. (**J**) IHC score of IFNβ and IFNα compared between pretreatment, post-niraparib R, and post-niraparib NR specimens, using the Wilcoxon rank-sum test with Benjamini-Hochberg correction. (**K** and **L**) Time-course quantification of SPP1 MFI (*n* = 4) following continuous IFN-γ (**K**) and IFN-β (**L**) treatment (10 ng/mL); solid lines represent continuous IFN dosing; dashed lines indicate SPP1 expression decay after IFN withdrawal. (**M**) SPP1 protein level in THP1 cells after continuous IFN (10 ng/mL) treatment with or without STAT1 inhibitor (fludarabine 2 μg/mL) for the indicated time points. *P < 0.05, **P < 0.01, ***P < 0.001.

To identify the principal regulators orchestrating this immunosuppressive network, especially in the multicellular ecosystems, we performed a systematic intercellular communication analysis using CellChat (30). This approach revealed SPP1 as the predominant signaling hub in posttreatment resistant tumors compared with sensitive tumors, with TAMs identified as the primary cellular source (Figure 5, A and B, and Supplemental Figure 7D). Ligand-receptor mapping further demonstrated augmented SPP1–CD44/integrin interactions that emanated from multiple cellular senders, including TAMs, myCAFs, eTregs, monocytes, pericytes, and endothelial cells (Supplemental Figure 7E). Differential gene expression analysis identified SPP1 as the most significantly upregulated transcript in resistant TAMs (Figure 5C), with modest yet consistent overexpression also seen across other stromal compartments (Figure 5D).

Although SPP1 RNA levels did not differ significantly between resistant lesions and pretreatment tumors in this limited scRNA-Seq cohort (Supplemental Figure 8A), longitudinal immunofluorescence assays of paired pre- and posttreatment specimens demonstrated that the abundance of CD68^+^SPP1^+^ TAMs increased slightly after niraparib exposure in responsive tumor, and showed pronounced elevation in resistant lesions (Figure 5E). These assays also confirmed a marked expansion of SPP1-expressing cells, particularly CD68^+^SPP1^+^ TAMs, in resistant versus responsive tumors (Figure 5E and Supplemental Figure 8B).

To further confirm the relationship between PARPi exposure and SPP1 upregulation, we engineered *Brca1/Trp53* double-knockout ID8 ovarian cancer cells using CRISPR/Cas9 (Supplemental Figure 8C). This genetically defined model recapitulates human HGSOC in which *BRCA* loss confers HRD and PARPi sensitivity, whereas *Trp53* ablation mimics the ubiquitous TP53 mutations characteristic of HGSOC. IHC analysis revealed a marked increase in SPP1^+^ cells within ID8 tumors after niraparib treatment (Figure 5F). Comparable results were obtained in HRD (*Brca*-deficient) E0771 breast tumors, in which PARP inhibition elicited SPP1 induction (Figure 5G). These findings indicate SPP1 plays a pivotal role in the therapy-induced TME remodeling.

During treatment, tumor cells in resistant lesions theoretically sustain persistent DNA damage, thereby continuously producing IFN (31, 32). Consistently, our earlier results demonstrated that the IFNG^+^ tumor cell niche was upregulated in posttreatment resistant lesions (Figure 3). Protein quantification further revealed that tumor cells exhibited the highest IFNG protein levels, which were maintained at high levels in resistant tumors (Figure 5H and Supplemental Figure 8D). IFN-γ protein levels in posttreatment lesions significant positively correlated with CD68^+^SPP1^+^ TAMs (Figure 5I). IHC revealed that IFN-β and IFN-α were also maintained at elevated levels in posttreatment resistant lesions (Figure 5J). We therefore stimulated the macrophage cell line THP1 with IFN. Flow cytometry and Western blotting demonstrated that IFN-γ, IFN-β, and IFN-α elevated SPP1 protein levels in a time-dependent manner, with SPP1 levels gradually decreasing upon withdrawal of IFN stimulation (Figure 5, K−M, and Supplemental Figure 8E). Moreover, STAT1 inhibitor blocked IFN-induced SPP1 protein upregulation (Figure 5M and Supplemental Figure 8F). In contrast, IFN stimulation of primary fibroblasts and the HGSOC cell line OV90 resulted in minimal SPP1 upregulation (Supplemental Figure 8, G and H). These results demonstrate that persistent IFN stimulation from tumor cells promotes SPP1 expression in macrophages within resistant lesions. Its multifaceted intercellular interactions within nonresponsive tumors position SPP1 as a potential therapeutic target for disrupting the immunosuppressive network.

### SPP1-mediated immunosuppression correlates with adverse prognosis after PARPi therapy.

**Figure 6:**
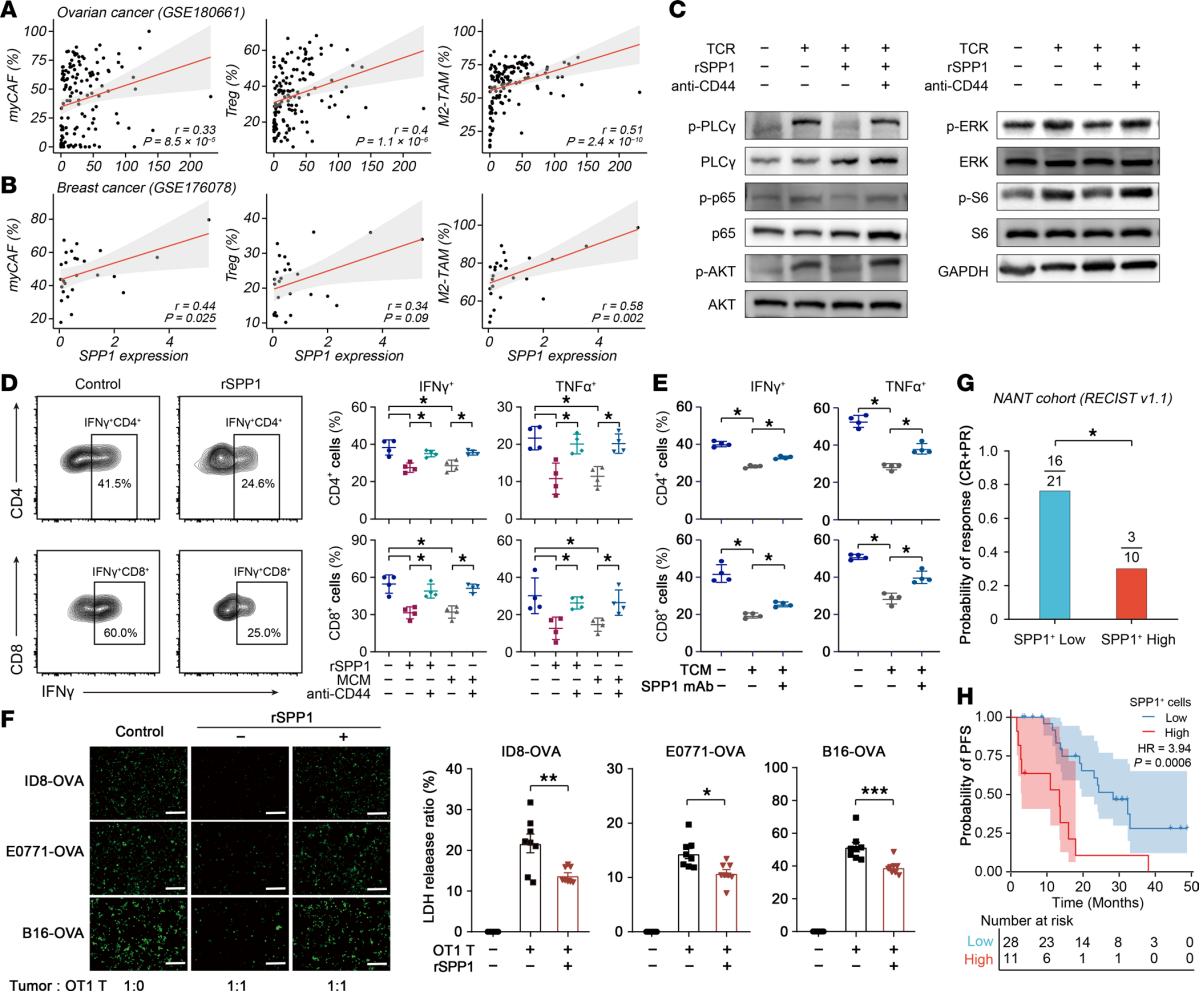
SPP1-mediated immunosuppression correlates with poor prognosis after PARPi therapy. (**A**) Spearman correlation analysis between global *SPP1* expression and proportion of M2-TAMs in macrophages, myCAFs in fibroblasts, and Tregs in CD4^+^ T cells in HGSOC using the GSE180661 scRNA-Seq dataset (*n* = 136). (**B**) Spearman correlation analysis (as in panel **A**) in breast cancer using the GSE176078 scRNA-Seq data set (*n* = 26). (**C**) Western blot analysis of the TCR signaling pathway activation in T cells stimulated with or without TCR, recombinant SPP1 (rSPP1), or anti-CD44 antibody. (**D**) Flow cytometry quantification of IFN-γ and TNF-α expression in CD4^+^ cells (upper) and CD8^+^ T cells (lower) after stimulation with or without rSPP1 or macrophage-conditioned medium (MCM), with or without anti-CD44 antibody (*n* = 4). Statistical significance was determined by Wilcoxon test with Benjamini-Hochberg correction. (**E**) Flow cytometry quantification of IFN-γ and TNF-α expression in CD4^+^ (upper) and CD8^+^ T cells (lower) treated with or without tumor-conditioned medium (TCM) and SPP1 mAb (*n* = 4). Statistical significance was determined by Wilcoxon test with Benjamini-Hochberg correction. (**F**) Representative images of residual ovarian tumor cells following co-culture with OT1 mouse-derived T cells (left). Lactate dehydrogenase (LDH) cytotoxicity assay results for different tumor cell lines (*n* = 8, right). (**G**) Clinical response rate (complete response, [CR] plus partial response [PR]) to niraparib monotherapy in the NANT cohort, stratified by low (*n* = 21) versus high (*n* = 10) density of SPP1^+^ cell infiltration. Statistical significance was determined by 2-sided Fisher’s exact test. (**H**) Kaplan-Meier estimates of progression-free survival (PFS) in the NANT trial, stratified by low (*n* = 28) and high (*n* = 11) density of SPP1^+^ cell infiltration. One patient was excluded from PFS analysis owing to loss of follow-up. Statistical significance was determined by log-rank test. *P < 0.05, **P < 0.01, ***P < 0.001. HR, hazard ratio.

To position SPP1 within the TAM-CN, we first interrogated its relationship with key components. Across the NANT HGSOC cohort (Supplemental Figure 9A) and the external scRNA-Seq datasets GSE180661 (HGSOC, Figure 6A) and GSE176078 (breast cancer, Figure 6B), *SPP1* abundance exhibited positive correlations with the relative frequencies of M2-polarized TAMs, myCAFs, and Tregs. Transcriptomic analysis further revealed a coordinated upregulation of *SPP1* and lineage-defining gene signatures for M2-TAMs, myCAFs, and Tregs in HGSOC, breast, and prostate cancers (Supplemental Figure 9B).

We next dissected the functional consequences of SPP1 signaling on T cell immunity. Assessment of key proximal and distal T cell receptor (TCR) signaling in human primary T cells showed that recombinant SPP1 (rSPP1) suppressed phosphorylation of PLC-γ, p65 (NF-κB), AKT, ERK, and S6, an effect abrogated by CD44 (the SPP1 receptor) neutralizing antibody (Figure 6C). rSPP1 and macrophage-conditioned medium markedly attenuated IFN-γ and TNF-α production in both CD4^+^ and CD8^+^ subsets (Figure 6D), and attenuated GZMB production in CD8^+^ subsets upon CD3/CD28 stimulation (Supplemental Figure 9C), both of which were blocked by CD44 neutralizing antibody (Figure 6D and Supplemental Figure 9C). Consistently, exposure to tumor-conditioned medium derived from HGSOC samples recapitulated this suppressive phenotype, whereas coadministration of an SPP1-neutralizing mAb (SPP1 mAb) restored cytokine output (Figure 6E). Using OT-1 transgenic T cells specific for ovalbumin, we further demonstrated that rSPP1 significantly impaired antigen-specific cytotoxicity against ovalbumin-expressing ID8 ovarian, E0771 breast, and B16 melanoma cells, as quantified by lactate dehydrogenase release (Figure 6F). Collectively, these data establish SPP1 as a conserved, multifunctional suppressor of antitumor T cell responses.

Given the observation of SPP1 enrichment within an immunosuppressive TME and its association with PARPi-resistant tumors, we next investigated whether baseline SPP1 abundance could prospectively stratify therapeutic outcomes. Within the NANT trial, patients whose tumors harbored high densities of SPP1^+^ cells had a markedly lower response rate to niraparib monotherapy (30% [*n* = 3 of 10] vs. 76.2% [*n* = 16 of 21]; *P* = 0.021) (Figure 6G) and a significantly shorter progression-free survival (PFS) (median 13.5 months vs. 28.3 months; *P* = 0.0006) (Figure 6H). Extending these observations, elevated *SPP1* expression was consistently associated with diminished overall survival in ovarian, breast, colorectal, lung, pancreatic, and hepatocellular carcinomas (Supplemental Figure 9D). Moreover, in patients with melanoma who were receiving anti-PD-1 or anti–CTLA-4 therapies, high tumoral *SPP1* levels predicted inferior clinical outcomes (Supplemental Figure 9E) (33, 34). Taken together, these data establish SPP1 as a biomarker of immunosuppression and a predictor of poor clinical outcomes following PARPi therapy.

### SPP1 blockade augments antitumor immunity and PARPi sensibility.

**Figure 7:**
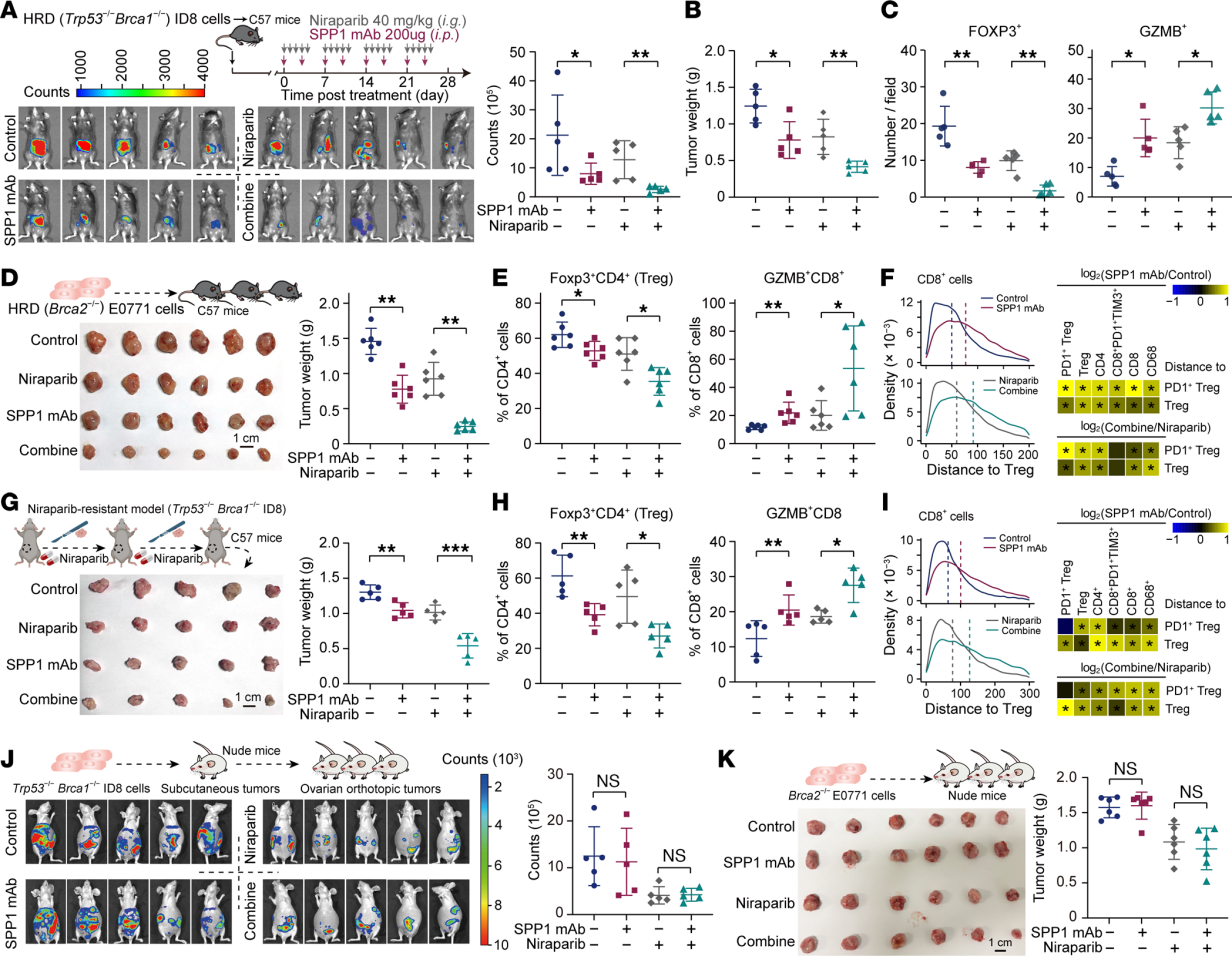
SPP1 blockade augments antitumor immunity and overcomes PARPi resistance. (**A**) Treatment schematic for orthotopic implantation of *Trp53^−/−^Brca1^−/−^* ID8 cells, followed by niraparib (i.g.; intragastric gavage) and/or SPP1 mAb (i.p.; intraperitoneal injection) administration. Right panel: Quantification of tumor bioluminescence at endpoint (*n* = 5 mice/group). (**B**) Surgically resected tumor weight of *Trp53^−/−^Brca1^−/−^* ID8 tumors at endpoint (*n* = 5 mice/group). (**C**) IHC quantification of intratumoral FOXP3^+^ and GZMB^+^ cells per field (×40 magnification; *n* = 5 mice/group). (**D**) Surgically resected tumor weight of *Brca2^−/−^* E0771 tumors at endpoint, with representative images (*n* = 6 mice/group). (**E**) Flow cytometry quantification of indicated T cell subsets in E0771 tumors among different groups at endpoint (*n* = 6 mice/group). (**F**) Nearest-neighbor distance analysis from indicated cells to Tregs or PD1^+^ Treg in *Brca2^−/−^* E0771 tumors. Vertical dashed lines represent the median distance; heatmaps compare control versus SPP1 mAb (upper) and niraparib monotherapy versus combination therapy (lower). (**G**) Schematic of niraparib-resistant ID8 tumor generation and surgically resected tumor weights at endpoint (*n* = 5 mice/group). (**H**) Flow cytometry quantification of indicated cells in niraparib-resistant ID8 tumors across treatment groups at endpoint (*n* = 5 mice/group). (**I**) Nearest-neighbor distance analysis in niraparib-resistant ID8 tumors (same format as panel **F**). (**J**) Bioluminescence imaging and quantification of orthotopic *Trp53^−/−^Brca1^−/−^* ID8 tumors in immunodeficient BALB/c-nu mice across treatment groups at endpoint (*n* = 5 mice/group). (**K**) Surgically resected tumor weight of *Brca2^−/−^* E0771 tumors from immunodeficient BALB/c-nu mice at endpoint (*n* = 6 mice/group). Statistical significance was determined by 2-tailed Wilcoxon rank-sum test (**A**−**E**, **G**, **H**, **J**, and **K**), *P < 0.05, **P < 0.01, ***P < 0.001, n.s. not significant; or Kolmogorov-Smirnov test (**F** and **I**), *P < 0.001.

Guided by the foregoing observations, we next evaluated the translational potential of SPP1-targeted therapy in combination with PARP inhibition. One week after orthotopic engraftment of HRD (*Trp53^−/−^Brca1^−/−^*) ID8 ovarian tumors into C57BL/6 mice, animals were randomized to receive niraparib with or without SPP1 mAb for 4 weeks. SPP1 mAb monotherapy produced a modest reduction in bioluminescence; however, the combination of SPP1 mAb and niraparib achieved synergistic tumor control, diminishing the bioluminescent signal by 88% (Figure 7A) and significantly lowering endpoint tumor weight relative to PARPi alone (Figure 7B). Mechanistically, IHC (Figure 7C and Supplemental Figure 10A) and flow cytometry (Supplemental Figure 10B) revealed that the combination regimen markedly depleted FOXP3^+^ Treg while simultaneously elevating IFN-γ and GZMB levels within tumor-infiltrating T cells. Notably, the combinational approach did not exacerbate hematologic toxicity, underscoring its preliminary safety (Supplemental Figure 10C). These findings were independently validated in HRD (*Brca*-deficient) E0771 breast tumors, where SPP1 mAb similarly augmented PARPi efficacy (Figure 7D), reduced Treg, PD1^+^ Tregs, and PD1^+^TIM3^+^CD8^+^-exhausted T cells, and increased IFN-γ, GZMB, and TNF-α levels in tumor-infiltrating T cells (Figure 7E and Supplemental Figure 10D). Spatial analysis revealed that SPP1 mAb treatment, either alone or in combination with niraparib, significantly increased the distance between various immune cell types (including CD68^+^, CD8^+^, CD4^+^, Tregs, and PD1^+^ Tregs) and Treg/PD1^+^ Tregs, indicating disruption of the Treg immunosuppressive niche (Figure 7F).

To interrogate the capacity of SPP1 blockade to reverse acquired PARPi resistance, serial in vivo passaging of *Trp53^−/−^Brca1^−/−^* ID8 tumors under continuous niraparib pressure generated a resistant model. In this setting, adjunct SPP1 mAb significantly enhanced the therapeutic response of PARPi (Figure 7G), concomitant with reduced Tregs, PD1^+^ Tregs, and PD1^+^TIM3^+^CD8^+^ T cells, and restored IFN-γ, GZMB, and TNF-α production (Figure 7H and Supplemental Figure 10E). Spatial analysis similarly demonstrated that SPP1 mAb administration significantly increased the distance between multiple immune cell types and Treg/PD1^+^ Tregs (Figure 7I). Critically, the therapeutic benefit of SPP1 mAb was abrogated upon orthotopic implantation of the ID8 (Figure 7J) or E0771 (Figure 7K) cells into immunodeficient mice, confirming that SPP1 blockade operates through an immune-dependent mechanism. Collectively, these data establish SPP1 as a central regulator of PARPi resistance that operates via immunosuppression, and they provide preclinical proof-of-concept for combining SPP1 antagonism with PARP inhibition to restore therapeutic efficacy.

## Discussion

Evidence has supported the synergy of immunotherapy and PARP inhibition, yet clinical combinations with immune checkpoint inhibitors have produced only incremental, rather than transformative, gains (11, 35). Leveraging the unique clinical framework of the PARPi monotherapy trial, which provides paired longitudinal specimens in treatment-naive patients without confounding cytotoxic exposure, we used a high-plex spatial technique (PCF), single-cell transcriptomics, and digital mIHC to resolve the TME remodeling. Our spatial protein profiling revealed 2 closely interacting cellular ecosystems: 1 dominated by IFN^+^ tumor cells and the other by immunosuppressive TAMs, both consistently enriched for Tregs and myCAFs. These CNs decreased in niraparib-responsive tumors, contributing to a favorable TME. Conversely, the IFN^+^ tumor cell–enriched neighborhood was significantly increased in resistant tissues after PARP inhibitor treatment, whereas the TAM-CN was maintained at proportions similar to pretreatment levels but underwent molecular and spatial alterations that shifted toward a more immunosuppressive phenotype. Mechanistically, persistent IFN release from residual tumor cells in resistant lesions promoted SPP1 expression in TAMs, thereby enhancing both intra-neighborhood interactions within the TAM-enriched niche and cross-neighborhood interactions between the TAM-enriched niche and the IFN^+^ tumor cell niche. Blockade of SPP1 (the central molecular mediator of this axis) remodeled both the cellular composition and spatial architecture of the tumor immune landscape and restored PARPi sensitivity. These results reveal how tumor cells actively remodel the microenvironment during resistance development and demonstrate that both tumor-intrinsic factors and the microenvironment collectively establish therapeutic resistance.

This study demonstrates that the elevated SPP1 level in resistant lesions most likely originates from IFN induction by persistent tumor cells. In responders, effective PARPi therapy triggers tumor cell death and a transient burst of IFN (31) that fleetingly upregulates SPP1 before subsiding as tumor cells are cleared. In contrast, resistant lesions sustain continuous DNA damage and ongoing IFN production throughout treatment (32), creating chronic stimulation that persistently elevates SPP1. These results suggest that the primary source and driving force of resistance reside within the tumor cell itself. However, this also highlights that the adaptive remodeling of the microenvironment by resistant tumors represents a critical component of resistance establishment, and targeting this adaptive response, such as by blocking the key mediator SPP1, can reverse resistance.

Interestingly, IFN exhibits a pronounced dual role in this remodeling process. Through activation of the JAK-STAT signaling pathway, promotion of antigen presentation, and enhancement of cytotoxic T lymphocyte, NK cell, and DC function, IFN serves as a central pathway for promoting immune activation and augmenting antitumor immunity (36, 37). On the contrary, chronic IFN signaling promotes immunosuppression—a concept now widely recognized. This immunosuppressive effect is mediated through multiple mechanisms, including induction of T cell exhaustion via upregulation of inhibitory receptors (PD-1, TIM-3, LAG-3), expansion of immunosuppressive myeloid-derived suppressor cells and regulatory T cells (18, 31, 38, 39). With regard to TAM, IFN serves as a traditional factor promoting M1-like, antitumor macrophage polarization (40). However, other studies and our scRNA-Seq data indicate that IFN-responding TAMs concurrently exhibit both high M1 and M2 signatures (41), whereas macrophage subsets with exclusively high M1 phenotype are absent in ovarian cancer. These findings align with the paradigm that the conventional M1/M2 dichotomy is insufficient to capture the full spectrum of macrophage plasticity and illustrate the dual role of IFN in macrophage programming (42). Our study further identifies an underappreciated mechanism by which chronic IFN, in the context of suboptimal therapeutic response, drives immunosuppression: promoting SPP1 expression in macrophages and thereby fostering the formation of an immunosuppressive niche.

Notably, IFNG^+^ tumor cells alone did not reach statistical significance when compared between resistant lesions and pretreatment samples, yet the IFN^+^ tumor cell–enriched CN showed significant differences, underscoring that individual cell types do not function in isolation. Rather, they form spatially structured functional communities where multicellular interactions are essential for biological effect, highlighting the critical importance of spatial analysis in uncovering clinically relevant mechanisms that remain obscured in conventional single-cell analyses (14, 43). Therapeutic perturbation introduces additional layers of microenvironmental complexity (44). Our present study demonstrates that after PARPi exposure, the TAM compartment becomes transcriptionally and functionally more homogeneous, with evidence for a more immunosuppressive phenotype and intensified intercellular crosstalk within resistant lesions. These adaptive alterations may explain why high baseline abundance of CNs in pre-therapeutic samples does not necessarily predict poor therapeutic efficacy, whereas posttreatment interaction of tumor cell–enriched CNs and TAM-CNs correlates with PARPi resistance. These findings underscore that spatially organized, multicellular communities and their adaptive response after therapy are key contributors to PARPi resistance.

However, the complexity of these networks suggests that interventions targeting single cellular constituents, such as the CSF-1R blockade for TAMs or anti-CD25 therapy for Tregs (11), are likely to achieve only partial efficacy. To address this challenge, our systematic analysis of single-cell data derived from this clinical trial has unveiled a dominant upregulation of secreted SPP1, which appears to orchestrate the TME in nonresponse lesions. Within these resistant tumors, SPP1 was notably elevated from TAMs, exerting effects on a panoply of cell types. Previous studies have proposed that SPP1 promotes tumor growth, metastasis, and chemoresistance across various malignancies through inducing epithelial-mesenchymal transition, autophagy, aberrant glucose metabolism, epigenetic alterations, and augmented drug efflux (45, 46). Furthermore, SPP1 is implicated in immunosuppression through its direct inhibitory effects on tumor-infiltrating lymphocytes and indirect enhancement of the immunosuppressive functions in myeloid-derived suppressor cells, TAMs, CAFs, and neutrophils (47–49). Although IFN may serve as an initiating factor, its dual immunostimulatory and immunosuppressive roles make it a suboptimal therapeutic target. Instead, neutralizing SPP1—a critical factor in the immunosuppressive arm of this balance—may represent a more appropriate strategy. Our data provide proof-of-concept for combining SPP1 antagonism with PARPi by showing that their combination significantly depleted Treg and enhanced PARPi sensitivity in HRD ovarian cancer, breast cancer, and PARPi-resistant models. Nevertheless, whether SPP1 or SPP1-high macrophages have additional tumor-intrinsic or microenvironment-independent roles in mediating PARPi resistance, and whether SPP1 can serve as a noninvasive serum biomarker for predicting and dynamically monitoring PARPi efficacy remains to be fully elucidated.

This study has several limitations. First, the scRNA-Seq cohort had a modest sample size, which we compensated for by integrating multiple platforms of sample analysis, functional validation, and mechanistic studies to ensure robust conclusions. Second, although it would be ideal to profile multiple tumor sites within each patient, tumor samples from the adnexa of late-stage HGSOC during diagnostic laparoscopies were frequently unavailable to ensure patient welfare and avoid additional trauma in this patient-centric study. Third, the NK marker (CD56) detection failed in our PCF panel due to technical issues, requiring future investigation of NK cell contributions. Fourth, because our stringent quality control required adequate tumor content in specimens, responder samples yielded fewer tissue cores than did nonresponder samples, which might introduce confounding factors. Fifth, whereas our data suggest high baseline SPP1 predicts poor response to PARPi therapy, it should be considered that SPP1 has been reported as a general prognostic biomarker in cancers and correlates with immune infiltration patterns (50, 51).

In summary, our study elucidates an underappreciated mechanism wherein chronic IFN signaling from persistent tumor cells orchestrates SPP1-mediated reprogramming of the TME, establishing spatially organized immunosuppressive niches that contribute to PARPi resistance. By integrating clinical trial specimens, high-resolution spatial profiling, and functional validation, we reveal baseline SPP1 as a clinically actionable biomarker and posttreatment SPP1 as a mechanistic linchpin. We anticipate that SPP1 blockade, particularly when combined with PARP inhibition, will offer a rational therapeutic strategy for HRD malignancies. This work underscores the necessity of targeting not only the tumor but also its immunosuppressive ecosystem to overcome resistance in the era of precision oncology.

## Methods

Please see Supplemental Methods for full materials and methods.

### Sex as a biological variable.

This study focused on HGSOC, which affects women. All patient samples were derived from female individuals, and all animal experiments used female mice to match the disease demographics. The findings may not be generalizable to male individuals.

### Patient cohort and human specimens.

This prospective study enrolled eligible female patients (aged 18–75 years) with newly diagnosed, histologically confirmed high-grade serous or endometrioid ovarian, peritoneal, or fallopian tube carcinoma deemed unresectable at baseline, as assessed by an upper abdominal CT score ≥3 or a laparoscopic Fagotti score ≥8. Paired FFPE tumor specimens were collected during baseline diagnostic laparoscopy for each participant. Patients with HRD (i.e., HRD-positive status), determined by a genomic instability score ≥42 or confirmed BRCA1/2 mutations; were enrolled in the single-arm, multicenter, phase 2 NANT trial (full eligibility criteria per NCT04507841, described previously; refs. 18 and 19); and received niraparib monotherapy (200 mg or 300 mg orally once daily) for two 28-day cycles prior to interval debulking surgery. Patients not enrolled in NANT (HRD proficient or declining participation) received standard neoadjuvant chemotherapy (paclitaxel-carboplatin) followed by surgery. Because this was a phase II efficacy/safety trial, pharmacokinetic analysis was not performed. All participants provided written informed consent before screening initiation, with study approval granted by the Research Ethics Commission of Tongji Medical College, Huazhong University of Science and Technology (approval 2020-S122). Fresh tumor tissue was immediately preserved in Miltenyi Biotec Tissue Storage Solution at 4°C until processing; each sample was divided into 3 portions: 1 submitted for central HRD testing (Precision Scientific), 1 enzymatically dissociated for scRNA-Seq, and 1 processed into FFPE blocks.

### Pathologic response evaluation.

Patient-level responses, such as RECIST 1.1, do not capture the heterogeneity within individual lesions (20). This occurs because RECIST 1.1 evaluates overall patient response across all lesions, whereas lesion-level histopathologic assessment focuses specifically on the biopsied specimen. Consequently, a patient may achieve partial response by RECIST criteria (overall tumor burden reduction) although the specific surgical biopsy site contains resistant residual disease that qualifies as pathologic nonresponse. Conversely, a patient with stable disease or progressive disease overall may undergo a biopsy that fortuitously captures a responding region. We therefore evaluated posttreatment specimens at the lesion level, using a modified histopathologic classification (21). Briefly, pathologic response was defined as complete absence of viable tumor or near-complete regression with only scattered single cells or nodules ≤2 mm. All other specimens, including those with predominantly viable tumor or only limited regression, were classified as pathologic nonresponse. Pretreatment analyses used patient-level clinical criteria; posttreatment analyses used this lesion-level histopathologic classification.

### Reanalysis of the GEO datasets.

We reanalyzed the GSE180661 (https://cellxgene.cziscience.com/collections/4796c91c-9d8f-4692-be43-347b1727f9d8), GSE176078, GSE155698, GSE137829, GSE141445, and GSE157703 (https://www.ncbi.nlm.nih.gov/geo/) datasets for external validation after noticing the correlations of the cell composition ratio among TAMs, eTregs, and myCAFs. Immune cells, including T cells and macrophages, inherited the clusters and annotations performed from the original research (29, 52–56). Fibroblast was extracted from all cells separately, followed by batch removing, clustering, and annotation. We calculated the M2 macrophage signature score, eTreg score, and myCAF score (57) in macrophages, T cells, and fibroblasts, respectively, to confirm the corresponding subclusters. Subsequently, the correlations of cell proportions among these 3 subclusters were evaluated.

### Statistics.

For analyses involving multiple groups, the adjusted *P* value (*P*adj) was calculated by performing the 2-tailed Wilcoxon rank-sum test followed by Benjamini-Hochberg correction to control the false discovery rate. Additional methodological details and figure legends pertaining to the statistical analyses are provided in the corresponding sections of the article. Significance was determined at the *P* or *P*adj < 0.05 level. All experiments were performed at least 3 times and the investigator was blinded to the group allocation during the experiments.

### Study approval.

The study approval was granted by the Research Ethics Commission of Tongji Medical College, Huazhong University of Science and Technology (approval 2020-S122).

### Data and code availability.

The raw PCF data supporting this study have been uploaded to the Zenodo repository (https://doi.org/10.5281/zenodo.15597290 and https://doi.org/10.5281/zenodo.15599108). scRNA-Seq data are available in the Gene Expression Omnibus (GEO) under accession number GSE222557. No original code was developed for this work. Supporting data values can be found in the Supporting Data Values file. All data supporting the findings of this study are available within the main text and supplemental materials.

## Author contributions

DL, KT, CX, WY, DM, YX, YF, and QG designed the study. QG led the clinical trial and oversaw sample allocation. WY, YX, KT, JL, JC, YL, KX, CC, SW, and DZ processed human specimens. JL and CX generated scRNA-Seq libraries. DL, X Liu, WP, QZ, CF and X Li performed data processing. DL, KT, CX, WY, YL, KX, JC, and CC performed experiment validation. DL, KT, GM, GNZ, DM, YX, YF, and QG wrote the manuscript with input from all authors.

## Conflict of interest

The authors have declared that no conflict of interest exists.

## Funding support

- National Natural Science Foundation of China (grants 82373332 and 82573663, to DL; 82403759, to CX; 82372928 and 82072889, to QG; 82441045 and 82272707, to YF).
- Key Program of Regional Joint Funds of the National Natural Science Foundation of China (grant U25A20117, to QG).
- National Key Technology Research and Development Program of China (grant 2022YFC2704200, to QG).
- Hubei Natural Science Foundation Outstanding Young Talents Project (grant 2022CFA051, to DL).
- Knowledge Innovation Program of Wuhan-Basic Research (grant 2023020201010051, to DL).
- nnovation Group Project of Hubei Province (grant 2023AFA036, to YF).
- Noncommunicable Chronic Diseases–National Science and Technology Major Project (grants 2025ZD0545600 and 2025ZD0545601, to YF).
- Hubei Science and Technology Major Plan Project (grant 2023BCA004, to DM).
- Zai Lab (Shanghai) Co., Ltd.

## Supplementary Material

Supplemental data

Unedited blot and gel images

Supporting data values

## Version 1 03/06/2026

In-Press Preview

## Version 2 04/15/2026

Electronic publication
