# Supplementary material for: Tumor cell–derived IFN spatially reprograms osteopontin-enriched macrophage niches to promote PARP inhibitor resistance
Source: J Clin Invest. 2026 Mar 6;136(8):e199035. doi: 10.1172/JCI199035 (PMC13078868; doi:10.1172/JCI199035)
Supplement: Supplemental data [file jci-136-199035-s178.pdf]

Supplemental Methods

Construction of Tissue Microarrays (TMAs)

Initially, formalin-fixed, paraffin-embedded (FFPE) tissue sections were subjected to H&E staining, followed by histopathological quality control by expert pathologists to exclude specimens with extensive necrosis, tissues containing little tumor or stromal components, or omental metastasis tissues predominantly composed of adipose tissue. Through rigorous quality control, 182 FFPE tissue blocks from 85 patients were ultimately included in this study, including 30 contralateral normal ovarian or fallopian tube tissues adjacent to cancerous lesions. From each block, 1–4 tissue cores (1.5 mm in diameter) were extracted and assembled into six tissue microarrays according to their volume and quality. The TMAs were sectioned at 3 μm thickness.

PhenoCycler-Fusion (PCF) of FFPE TMAs

Sections were baked at 55°C for 1 hour, followed by deparaffinization with Histochoice Clearing Agent (PN# H103-4L; VWR) and rehydration through a graded ethanol series. Heat-mediated antigen retrieval was performed in Tris-EDTA buffer (pH 9), after which the sections were equilibrated to room temperature and transferred to Hydration Buffer from the PhenoCycler-Fusion Sample Kit (#7000017; Akoya Biosciences).

Commercially available PhenoCycler antibodies conjugated with oligonucleotide barcodes (Akoya Biosciences) were diluted with buffer (#7000017; Akoya Biosciences) at optimal concentration to prepare the first antibody cocktail (containing 39 antibodies). Later, slides were pre-treated with a pre-blocking solution made of N, J and S blockers in Staining Buffer. Then cocktail was applied to the sections and incubated overnight at 4°C. The following day, the sections were underwent rinsed with Staining buffer and followed by three rounds of fixation with paraformaldehyde (PFA; PN# 15710; Electron Microscopy Sciences), methanol, and PhenoCycler Fixative reagent, followed by storage in 4°C storage buffer until imaging.

Following the PhenoCycler-Fusion User Guide, reporter working solution was prepared, and the flow cell (#240204; Akoya Biosciences) was assembled onto the slides. Each imaging cycle included three reporters. Blank cycles (containing reporter stock solution without fluorescent reporters) were performed at the beginning and end of each run to subtract autofluorescence background. The slide was transferred to the flow cell carrier and imaged on a PhenoCycler-Fusion system (Akoya Biosciences) with the following exposure settings: DAPI—1 ms, ATTO550—150 ms, AF647—150 ms, and AF750—150 ms. Integration of the PhenoCycler automated fluidics cycler with the PhenoImager Fusion system enabled fully automated reporter hybridization, imaging, and dehybridization, capturing whole-slide images of three markers (+DAPI for nuclear staining) per cycle. Images were exported in QPTIFF format.

PCF data pre-processing and cell segmentation

Final QPTIFF files were processed using QuPath software (v0.3.2) for data pre-processing and cell segmentation. Prior to analysis, each image underwent quality control via visual inspection across the entire slide. Marker signal intensity, staining specificity, and artifacts (e.g., out-of-focus regions, tissue folds, debris) were manually annotated and excluded. Nuclear segmentation was performed using StarDist (v0.3.2), a deep learning-based method with default parameters. Cytoplasmic segmentation was derived by morphologically dilating nuclear masks by 5 μm. The centroid of each cell was determined using the x-y coordinates of the nuclear object's centroid. Segmentation quality was validated for each slide, demonstrating consistent and reliable results.

PCF quality control

QuPath generated a matrix file containing spatial coordinates and protein expression data for 4,977,260 cells. For each marker, the mean whole-cell fluorescence intensity was extracted for analysis. Low-quality cells (total intensity <10 or >2000 across all markers) were excluded.

Outlier thresholds were set for individual markers to filter extreme intensities. Each TMA dataset was processed independently using the Seurat standard workflow: data scaling, PCA (based on 19 cell type markers: CD45, CD20, CD79a, CD38, CD3e, CD4, CD8, CD68, CD14, HLA-DR, CD11c, MPO, CD31, CD34, Podoplanin, Vimentin, αSMA, PanCK, EpCAM), UMAP dimensionality reduction, and unsupervised clustering. Clusters lacking expression of any marker or exhibiting aberrantly high signals of all markers were removed as unstained cells or artifacts. Additionally, 13 tissue cores with high autofluorescence were manually excluded from downstream analysis (TMA1_P_2, TMA2_P_2, TMA3_A_1, TMA3_E_4, TMA4_A_1, TMA4_B_1, TMA4_B_2, TMA5_O_7, TMA5_P_7, TMA5_I_7, TMA6_G_7, TMA6_J_7, TMA6_K_7).

PCF definition of cell types

Post-quality control, reciprocal PCA (RPCA) anchor-based integration was applied to adjust for technical and biological variations across TMAs. The standard workflow (scaling, PCA, UMAP, clustering) was repeated using the 19 markers. Major cell types were annotated based on cluster-specific marker expression: 1) B cells: CD45, CD20, 2) plasma cells: CD79a, CD38, 3) T cells: CD45, CD3E, 4) TAM and DC: CD45, CD14, CD68, 5) Neutrophil: CD45, MPO, 6) endothelial cells: CD31, 7) fibroblasts: Vimentin, αSMA, 8) epithelial cells: PanCK, and EpCAM. Cluster resolution was optimized to maximize separation by these markers. Two additional refinements were made: 1, Lymphatic endothelial cells ($CD31^{+}Podoplanin^{+}$) were identified by re-clustering Podoplanin-high populations; remaining $CD31^{-}$ cells were classified as fibroblasts. 2, Epithelial-T cell mixtures were resolved via secondary clustering. Annotations were validated by revisiting original images. Subpopulation identification followed the same workflow, using subtype-specific markers (Supplemental Figure 2). Additionally, 9 tissue cores with epithelial cell proportions exceeding 90% and 22 pre-treatment cores exhibiting epithelial cell proportions below 5% were excluded from subsequent compositional analyses.

PCF calculation of CN and PCI

Cellular neighborhoods (CNs) (27) were defined as a 25 μm-radius window around each index cell. Windows with <5 cells (including the index) were excluded (9.96% of cells). Cell proportions within each window were clustered using MiniBatchKMeans, with optimal cluster numbers determined by the elbow method. CNs were labeled by their most abundant cell type.

For pairwise cell–cell interaction (PCI) (22), the five nearest neighbors to each index cell were counted and annotated. Interactions between cell types *i* and *j* were quantified using the formula:

$$\mathrm{PCI_{ij}} = \log_{10}\left(\frac{\mathrm{N_{ij}} \cdot \mathrm{N_t}}{\mathrm{N_i} \cdot \mathrm{N_j}} + 0.0001\right)$$

where N_{ij} = interactions between *i* and *j*; N_i and N_j = total interactions involving i or j; N_t = total interactions of all cell types.

Single-cell RNA-Seq data processing and annotation

Single-cell RNA-Seq data (GSE222557) were processed as we recently described (18). In brief, Kallisto-bustools was used to calculate UMI counts for each droplet, and the FASTQ files were referenced to the GENCODE v37 human genome. The kallisto-bustools workflow was set to 'standard' to retain only exonic reads. Seurat was used for normalization, identification of highly variable genes, regression analysis, standardization, principal component analysis (PCA), Uniform Manifold Approximation and Projection (UMAP) dimensionality reduction visualization, and Louvain clustering, with further quality control in UMAP space. After preprocessing and quality control of each sample, we additionally utilized reciprocal canonical correlation analysis (RPCA) to remove sample-level batch effects after principal component analysis (PCA) and before UMAP dimensionality reduction. Cells were annotated into nine major cell types: 1) B cells (MS4A1, CD79A), 2) epithelial cells (EPCAM, KRT18), 3) endothelial cells (VWF, PECAM1), 4) fibroblasts (COL1A1, DCN), 5) mast cells (TPSB2, CPA3), 6) myeloid cells (CD14, AIF1, LST1), 7) plasma cells (MZB1, DERL3), 8) pericyte

(RGS5, COL18A1), and 9) T cells and NK cells (CD2, CD3D). Extensive literature review facilitated the further annotation of sub cell types.

When comparing the cell composition ratios, we used the average values of data points from multiple sites in the same patient. Raw data from individual samples are retained, for example, for use in paired per- and post-treatment analyses.

Graph-based differential abundance testing

To explore the differences in cell quantity in different cell states independently of clusters, we performed differential abundance testing analysis with the miloR package. Specifically, neighborhoods were first defined by sampling a subset of representative cells on a k-nearest neighbor (KNN) graph. The numbers of cells contained in each neighborhood were then counted. Finally, the negative binomial generalized linear model framework was used to compare the abundance of cells in different states in each neighborhood.

Cellular Communications

Based on the single-cell gene expression matrix, we performed the matrix on CellChat v2.1.2 to analyze the ligand-receptor interactions. We obtained secreted signaling and cell-cell contact interaction information from the CellChatDB database and infer the communication network among different cell types. To compare cell-cell interaction between the two conditions (response vs non-response), the above workflow was run separately for each condition. Following this, two separate CellChat objects were obtained and merged for additional analysis.

Gene set enrichment analysis (GSEA)

Using the presto package v1.0.0, we calculated the differential genes between samples from response and non-response samples. The genes were ranked in descending order based on their AUC values, and those with avgExpr > 0.05 were selected. GSEA analysis was performed on the list of AUC values of these genes using the clusterProfiler package v4.2.2. The gene sets from the molecular signatures database were collected using the msigdbr package v7.5.1.

Gene set variation analysis (GSVA)

The AverageExpression function from the Seurat package was used to generate a pseudobulk matrix. Subsequently, the clusterProfiler package v4.2.2 was used to extract gene sets from the Human Molecular Signatures Database (MSigDB) database. The GSVA package v1.52.3 was employed to get gene sets expression matrix. Based on the GSVA results, we evaluated the impact of treatment on pathway variance (Gini index, Entropy, Atkinson index, Kolm index, Ricci-Schutz index, Theil index, and coefficient of variation) using the ineq package v0.2-13. The $MMP9^{+}$ TAMs and $MARCO^{+}$ TAMs were excluded from this analysis for their low cell numbers in the post-treatment groups and proliferating TAMs were excluded for the cell cycle nature of the macrophage subtypes.

Cells

OV90, THP-1 and E0771 cell lines were purchased from ATCC and OVCAR8 was obtained from MDACC's Characterized Cell Line Core. ID8 cells were bought from Sigma-Aldrich (Cat#SCC145). THP-1and OVCAR8 cells were cultured with RPMI 1640 medium with 10% FBS (Cellmax, sa211). ID8 and E0771 cell were cultured in DMEM (Cellmax, CGM103) with 10% FBS. OV90 cells were cultured with MCDB 105/199. All cell lines were passaged less than 20 times. Cells used in all experiments were routinely checked for mycoplasma contamination (Mycoalert Mycoplasma Detection Kit, Lonza).

Animal

Female C57 BL/6, BALB/c-nu mice were purchased from GemPharmatech (Nanjing, China) and all animal experiments were approved by the Committee on Ethics of Animal Experiments of Tongji Medical College. *Trp53*, *Brca1* and *Brca2* were knocked out in ID8 or E0771 cell line using CRISPR/Cas9, then the knockout of the genes was verified using Sanger sequencing, as we recently described (18). In the xenograft models, about $1x10^{6}$ $Trp53^{-/-}$ $Brca1^{-/-}$ ID8 cells were orthotopically injected into the ovaries of 4-weeks-old female C57BL/6 mice. For

E0771 breast cancer model, about $5x10^5$ *Brca2*$^{-/-}$ E0771 cells were orthotopic injected into the mouse mammary gland. When tumors reached about 100 mm^3, the mice were randomly assigned to four groups (a. control group, b. niraparib group, c. anti-SPP1 neutralizing antibody group, d. niraparib combined with anti-SPP1 neutralizing antibody group). Niraparib (Selleck, MK-4827) was given once a day by intragastric administration (40mg/kg) and anti-SPP1 antibody (Bioxcell, BE0382) was given twice a week via intraperitoneal injection (200μg/mouse). For control group, mice were given resolvent orally and sterilized PBS for intraperitoneal injection.

Niraparib-resistant ID8 tumor model

Firstly, *Trp53* and *Brca1* double-knockout ID8 cells (1×10^6) were orthotopically injected into the ovaries of C57BL/6 mice. Once the tumor formed (about 1 week), continuous niraparib administration (oral gavage, 50 mg/kg/day) was initiated. Upon reaching a predefined tumor volume, mice were sacrificed and tumors were aseptically excised, minced into 1-2 mm^3 fragments, and surgically re-implanted into the ovarian bursa of naive recipient mice under sterile conditions. With concurrent niraparib treatment maintained throughout subsequent passages, niraparib-resistant tumors were attained and subjected to later experiments.

Multiplex immunohistochemistry (mIHC)

As soon as the clinical sample arrived at the laboratory, a portion of the tumor tissue was used for paraffin embedding and further immunohistochemistry. H&E slide of each sample was used to assess the quality of ovarian cancer tissue sections. An optimal slide should contain a proper proportion of tumor and interstitial components, and only the sections that achieved this criterion were subjected to the following multiplexing staining. The mIHC experiment was performed using a Tyramide signal amplification (TSA) system according to the manufacturer's instruction (Opal 6-Plex Manual Detection Kit, NEL81100KT, Akoya Biosciences, America). The primary antibodies used in mIHC are presented in

Key_Resources_Table. After DAPI staining, slides were covered with coverslips and scanned by PHENO IMAGER^{HT} (Akoya Biosciences, America).

mIHC image analysis

The entire fluorescence sections were firstly scanned by a Vectra 3 multispectral imaging platform (Akoya Biosciences) at 4X magnification. Phenochart (Akoya Biosciences) was used for further manual annotation of tumor and interstitial regions. For the conjunctive analysis of the three marker panels, the matching fields of the serial section were obtained for each sample. To avoid staining artifacts, tissue edges were excluded from our analysis. The annotated regions were then scanned at 20X in multispectral fashion. Cell segmentation was performed using inForm software (v2.6.0 Akoya Biosciences/PerkinElmer) to achieve an optimal nuclear segmentation, by iteratively adjusting the DAPI intensity, minimum nuclear size, splitting factor and other parameters. The data were further analyzed using the phenoptr v0.3.2 package for R software. We used a semi-artificial method to determine the threshold for each marker that showed stronger accuracy and stability. Thresholds for both the mean pixel intensity and the total pixel intensity for all channels were manually set for each slide and evaluated by four independent researchers. The specificity and sensitivity of the positive threshold were further examined across all fields of views (FOVs) using a visualization strategy where the positive cells for each marker were superimposed over the spectral unmixing image of the corresponding channel. If poor classification persisted, the above threshold adjustment and visual inspection would be repeated until the final binary classification result satisfied a pathologist.

Dual-immunohistochemistry

The dual-immunohistochemistry was performed by using a mIHC kit (RS0039, Immunoway, China) under the manufacturer's instruction. Three panels (CD68/SPP1, FAP/SPP1, CD3/SPP1) were conducted respectively in our analysis. The slides were successively incubated with

CD68/FAP/CD3 antibody and SPP1 antibody at 4 °C overnight. Finally, slides were scanned using a Pannoramic MIDI (3DHISTECH, Hungary). After scanning, the results were reviewed by Case Viewer 2.3 software. For every slide, five randomized fields were captured under 40X magnification. Then the number of DAPI and indicated cell types were respectively calculated in Image J software. The cells in five fields were summed up in the later analysis. The percentage of one cell was calculated as follows: the number of one cell/DAPI number × 100%.

Immunohistochemistry (IHC)

The tumors were washed with PBS and fixed with 4% paraformaldehyde when the mice were sacrificed. Then tumors were embedded with paraffin for IHC analysis. Slides were de-waxed, soaked in gradient ethanol and treated with 3% H_2O_2 to wipe off endogenous peroxidase. Then antigen retrieval was performed according to the direction of primary antibodies. All primary antibodies were incubated overnight in our cryogenic laboratory and HRP-conjugated secondary antibody was incubated at room temperature for an hour. The targets were stained with DAB and slides were scanned by GScan-40 (Guangying Technology, China). The cells stained by DAB were deemed to be positive. Four randomized fields were captured at 40X magnification for each slide and the average positive dots of the four fields represented the expression level of the section.

T cell cytotoxic assay

The splenic tissue from the OT1-mouse was subjected to mechanical homogenization and subsequent filtration through a 70-micrometer mesh to obtain a suspension of single cells. To eliminate erythrocytes, the resulting cell suspension was treated with a red blood cell lysis buffer. T lymphocytes were then isolated using a Mouse T Cell Enrichment Kit (Thermo Fisher Scientific, catalog number 8804-6820). ID8-OVA, E0771-OVA, and B16-OVA tumor cell lines were labeled with CFSE and seeded into 96-well microplates at a density of 10,000 cells per well. Once full adherence was achieved, approximately 10,000 T cells derived from the OT1

mouse were introduced to interact with the tumor cells in the presence of varying concentrations of mouse recombinant SPP1 (TargetMol, catalog number TMPK-00807). Cytotoxicity was assessed by measuring lactate dehydrogenase (LDH) release, in accordance with the manufacturer's protocol (Sigma Aldrich, 11644793001), 24 hours post-co-culture. Concurrently, the 96-well microplates were rinsed with PBS to remove non-adherent or lysed tumor cells. The remaining cells were then photographed using an inverted fluorescence microscope.

Flow cytometry

As soon as xenograft tumors were excised, a part of them were washed with chilled PBS and used for flow cytometry analysis. Firstly, the tumors were shredded into small particles and digested with mouse tumor dissociation kit (Miltenyi Biotec, catalog: 130-096-730) for 45 minutes. The mixture was passed through 40 μm SmartStrainers (Miltenyi Biotec) to get single cells. Then the single cells were planted into 24-well plate and stimulated with cell activation cocktail (Biolegend, 423304) for 4 hours. The cells were washed twice and incubated with Zombie NIR™ Fixable Viability Kit (BioLegend,423106) for 15 minutes. Then the cells were subjected to cell membrane staining and intracellular staining in sequence. Cells were permeabilized by Fixation/Permeabilization Solution Kit (BD bioscience, 554714) before intracellular staining. Finally, cells were washed with PBS and examined by a Beckman Coulter Cytoflex.

For human PBMCs were attained from healthy donors, the T cells were sorted by anti-CD3 magnetic beads (Miltenyi Biotec, Germany, Lot no: 5230307554) and stimulated by human CD3/CD28 T cell activator (STEM CELL, Catlog #10791). T cells were seeded in 96 well-plates and treated with human recombinant SPP1 (Novoprotein, C544) and tumor conditioned medium with or without SPP1 mAb. Following a three-day incubation period, the T cells were harvested and prepared for flow cytometry analysis.

For SPP1 detection, THP1, CAF, and OV90 cells were harvested and washed twice with ice-cold PBS. Cells were stained with a fixable viability dye, followed by fixation and permeabilization using the BD Cytofix/Cytoperm™ Fixation/Permeabilization Solution Kit (BD Biosciences, Cat# 554714). Subsequently, the cells were incubated with an anti-SPP1 antibody at 4°C for 30 minutes.

Analysis of T cell signaling pathways

Human T cells were sorted from PBMCs and activated with anti-CD3/anti-CD28 activator for 48 hours. Subsequently, the activator was removed, and the cells were cultured in a complete medium supplemented with IL-2 for an additional 4 days to obtain rested effector T cells. For signaling analysis, these rested T cells were re-stimulated with TCR activator in the presence or absence of recombinant SPP1 (Novoprotein, C544) or neutralizing anti-CD44 antibody (Leinco, C382) for 2 hours. Cells were immediately harvested on ice for protein extraction.

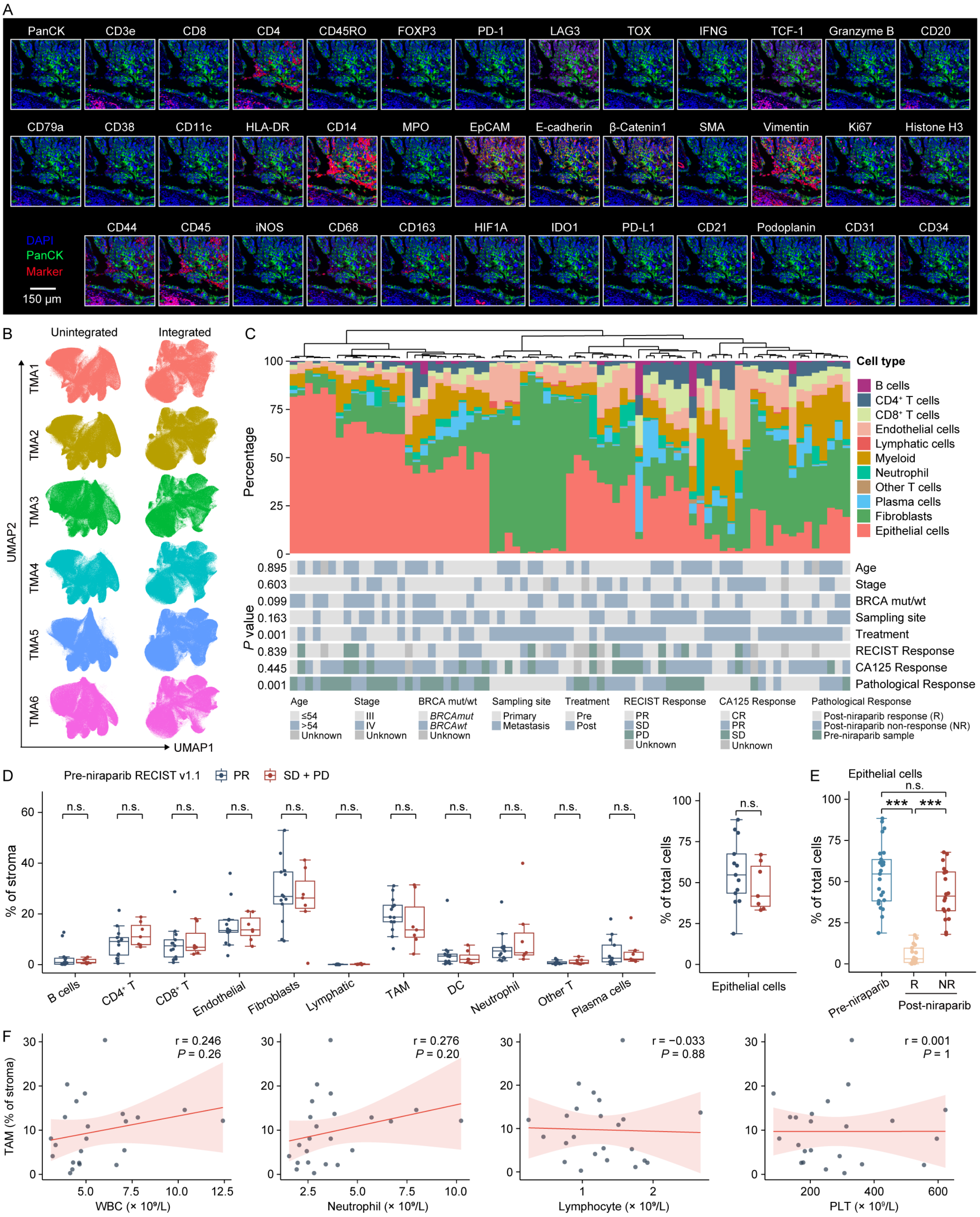

Supplementary Figure 1. Quality control and quantification of major cell types using PhenoCycler-Fusion (PCF) spatial technology.

(A) Representative fluorescence images validating antibody specificity. Nuclear staining (DAPI, blue), epithelial marker PanCK (green), and corresponding markers (red) are shown. CD56 was excluded due to failure of detection. **(B)** Batch-specific UMAP projections of PhenoCycler-Fusion data (n = 6 TMAs) before and after integration. **(C)** Hierarchical

clustering of cell type proportions, using the mean value of multiple tissue cores obtained from the same paraffin mass (upper, n = 73), with integrated clinical metadata (lower). **(D)** Quantitative analysis of cell type proportions in niraparib pretreatment samples, stratified by clinical response categories: PR (n = 13) versus SD + PD (n = 7). **(E)** Comparison of epithelial cell proportions across treatment phases and response categories. Pre-treatment (n = 25), post-niraparib responders (R, n = 21), and post-niraparib non-responders (NR, n = 18) were analyzed. **(F)** Correlation analysis of TAM levels and peripheral blood cell counts (n=23).

Statistical significance was determined by Permutational Multivariate Analysis of Variance (PerMANOVA) (**C**), two-tailed Wilcoxon rank-sum test with Benjamini-Hochberg correction (**D and E**), or Pearson correlation analysis (**F**). ***p.adj < 0.001, n.s. not significant.

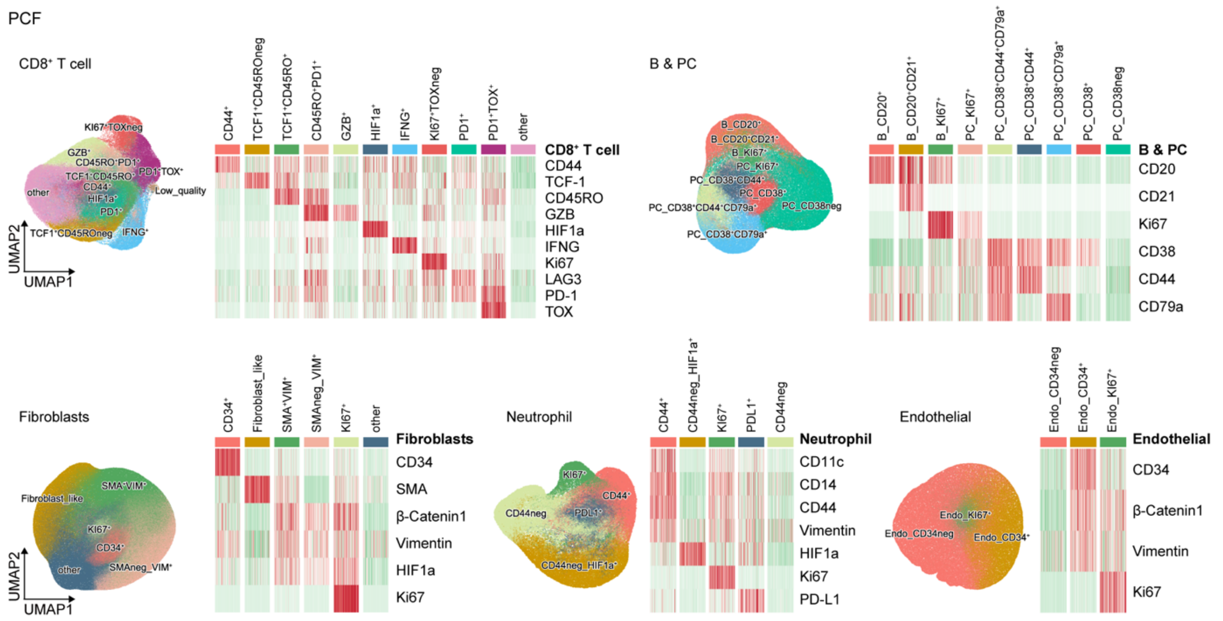

Supplementary Figure 2. Cell subtype annotation using PCF.

Integrated UMAP visualization with heatmap showing color-coded cell subtype annotation of CD8$^+$ T cells, B & PC, Fibroblasts Neutrophil and Endothelials derived from PCF analysis; corresponding heatmap displays the z-scored marker expression confirming lineage-specific signatures.

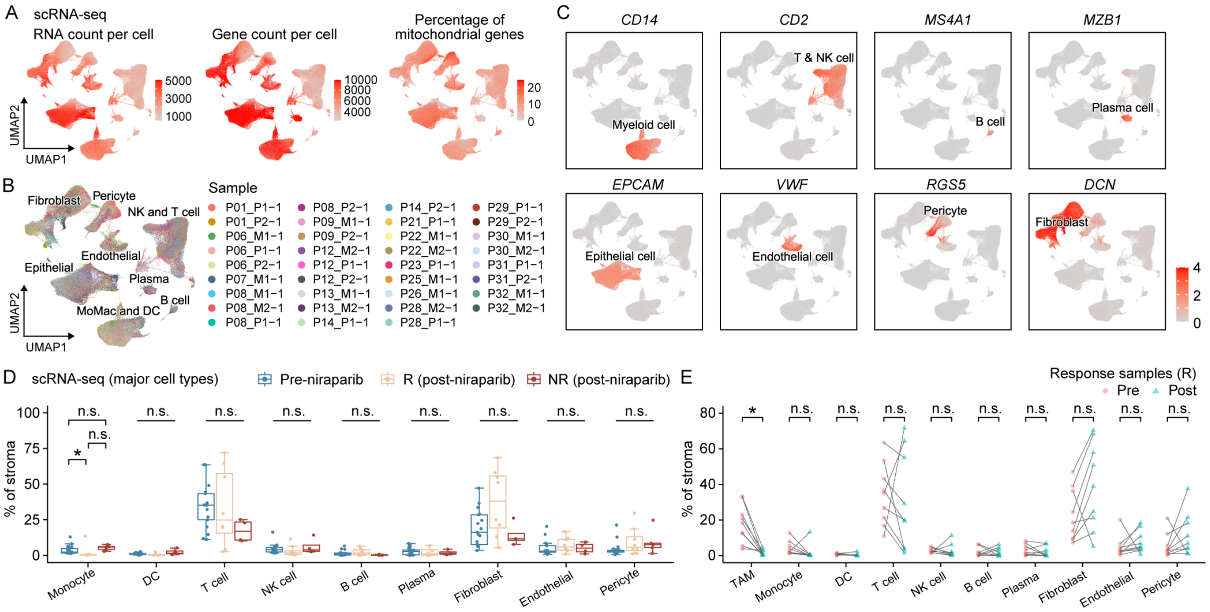

Supplementary Figure 3. Annotation and quantification of major cell types using scRNA-seq.

(A) Quality control metrics showing UMAP embeddings colored by total unique molecular identifier (UMI) for RNA count, gene count, and mitochondrial RNA proportion. **(B)** Sample integration assessment showing batch-aware UMAPs colored by sample ID, demonstrating minimal batch effects post-integration. **(C)** Cell type identity confirmation showing UMAP plots of major cell clusters colored by expression of selected lineage-specific marker genes (color gradient represents normalized expression). **(D-E)** Quantification of cellular composition of major cell types from scRNA-seq data. Comparisons among pre-treatment (n=17), post-niraparib responders (R, n=8) and post-niraparib non-responders (NR, n=5) using Wilcoxon rank-sum test (**D**). Paired comparison between post-niraparib responders and pre-treatment tumors (n=9 pairs) using paired t-test (**E**). Statistical significance was assessed by two-tailed tests with Benjamini-Hochberg correction; adjusted p-values (p.adj) are reported. *p.adj < 0.05, n.s. not significant.

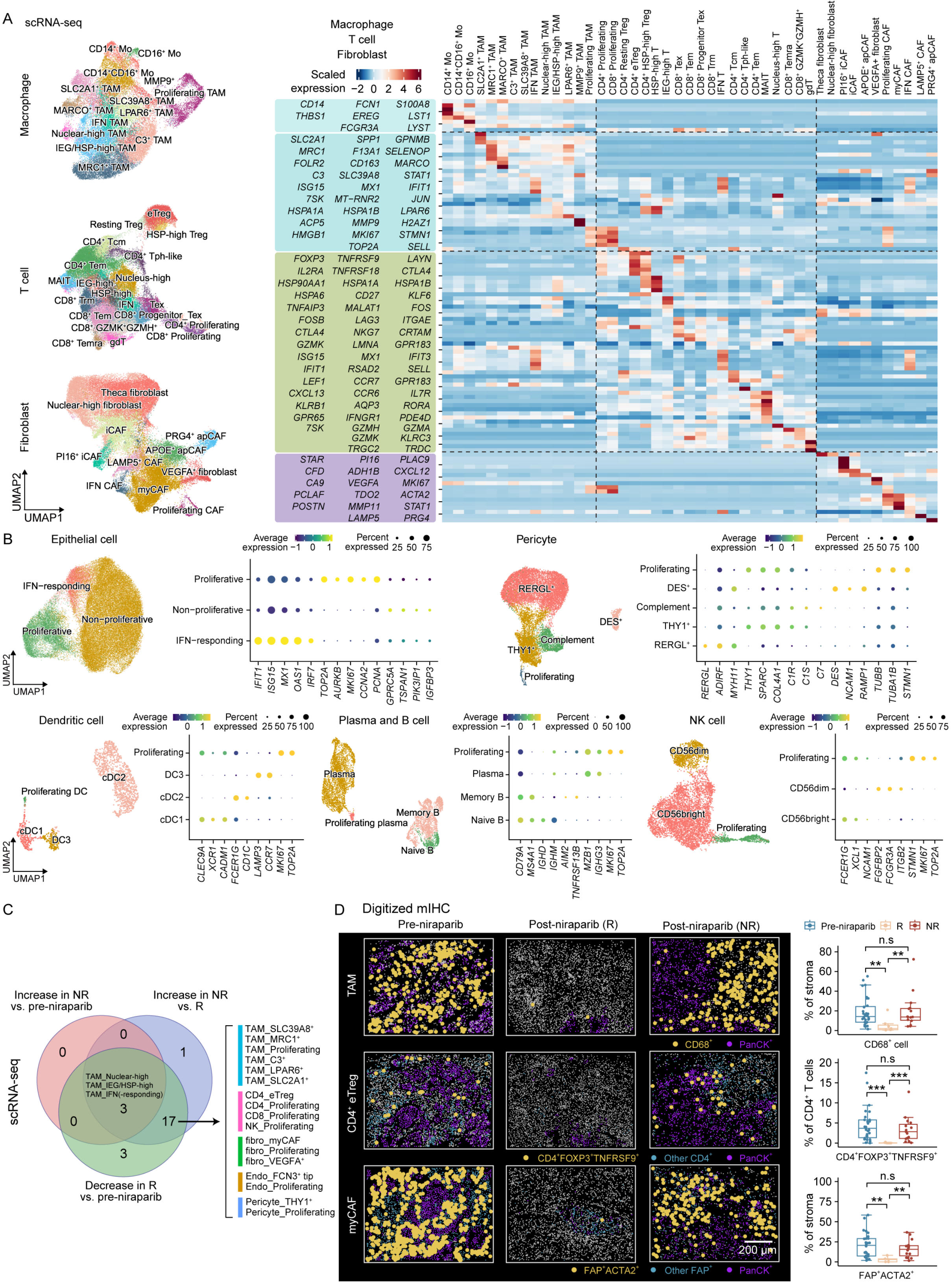

Supplementary Figure 4. Persisted TAMs, eTregs, and myCAFs in niraparib-resistant tumors.

(A) UMAP visualization of cell clusters (left) and heatmaps of average expression for selected marker genes (right) across macrophage, T cell, and fibroblast subtypes. **(B)** UMAP

visualization of cell clusters and bubble plots of selected marker genes for epithelial, pericyte, plasma/B, NK, and DCs subtypes. **(C)** Venn diagram of cell subtypes (scRNA-seq) that increased in non-responsive lesions (NR vs. Pre; NR vs. R), while decreased in responsive lesions vs. pre-treatment. **(D)** Representative digitized phenotypes of TAMs ($CD68^{+}$), eTregs ($CD4^{+}FOXP3^{+}TNFRSF9^{+}$), and myCAF ($FAP^{+}ACTA2^{+}$) (left). Quantification of the differences in cell proportion across treatment phases and response categories. (right). Wilcoxon rank-sum test (Pre-treatment, n=27-32; R, n=5-7; NR, n=15). *$P < 0.05$, **$P < 0.01$, ***$P < 0.001$.

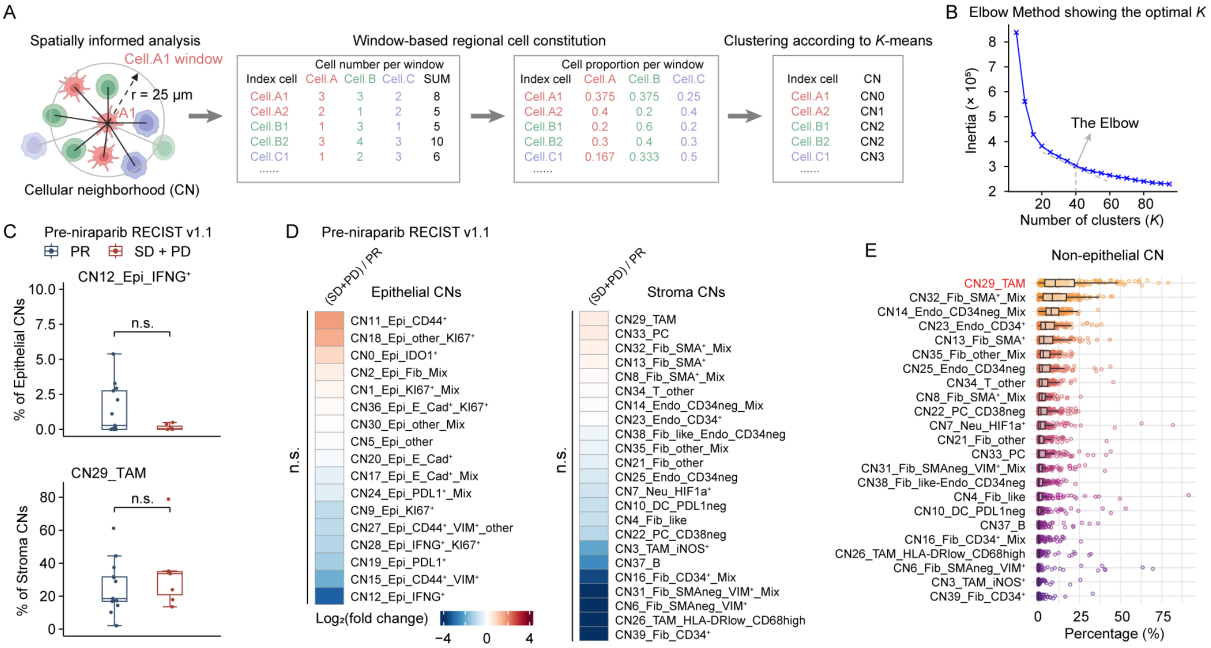

Supplementary Figure 5. No significantly differences in baseline cellular neighborhood composition between different responses to niraparib.

(A) Algorithmic framework for cellular neighborhood (CN) detection. **(B)** Identification of optimal clustering numbers using the elbow method. **(C)** Quantification of CN12_Epi_IFNG^{+} proportion within epithelial CNs and CN29_TAM proportion within stroma CNs, comparing RECIST v1.1 responders (PR, n=13) versus non-responders (SD+PD, n=7). **(D)** Heatmaps showing Log2-fold changes in abundance of epithelial CNs (left) or stroma CNs (right) between RECIST v1.1 responders (PR, n=13) versus non-responders (SD+PD, n=7). **(E)** CN-specific contributions to stromal regions across all samples. Statistical significance was determined by two-tailed Wilcoxon rank-sum test with Benjamini-Hochberg correction (**C** and **D**). n.s. not significant.

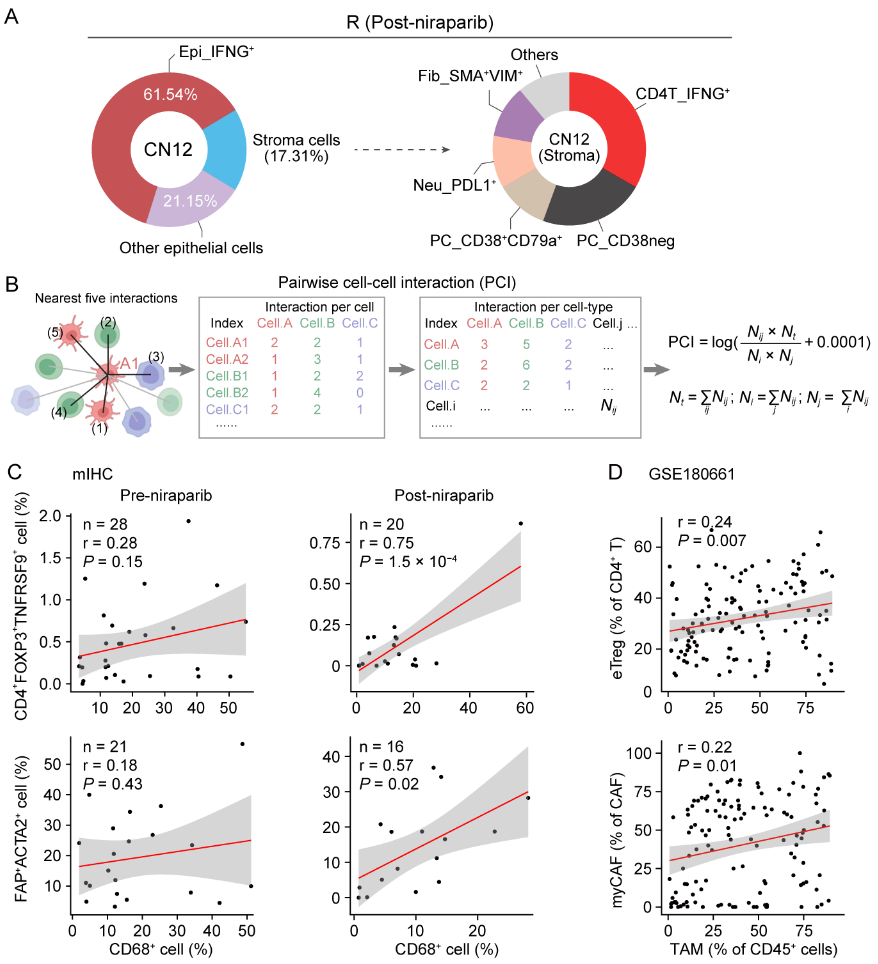

Supplementary Figure 6. Validation of co-variation of TAMs, eTregs, and myCAFs.

(A) Donut plots illustrating the cellular constituents of CN12_Epi_IFNG^{+} in post-niraparib responder (R) specimens. **(B)** Computational framework for quantifying pairwise cellular interactions (PCI) using PCF spatial technology. **(C)** Correlation between the proportion of the indicated cells over stromal cells across pre-treatment (left) and post-niraparib tumors (right), based on mIHC. Pearson correlation analysis. **(D)** External validation using HGSOC GSE180661 scRNA-seq data: correlation analysis of the proportion of myCAF in CAFs, eTreg (sum of original Treg1, Treg2, and Treg3) in CD4^{+} T cells and the proportion of TAMs in CD45^{+} cells (n = 130). Pearson correlation analysis.

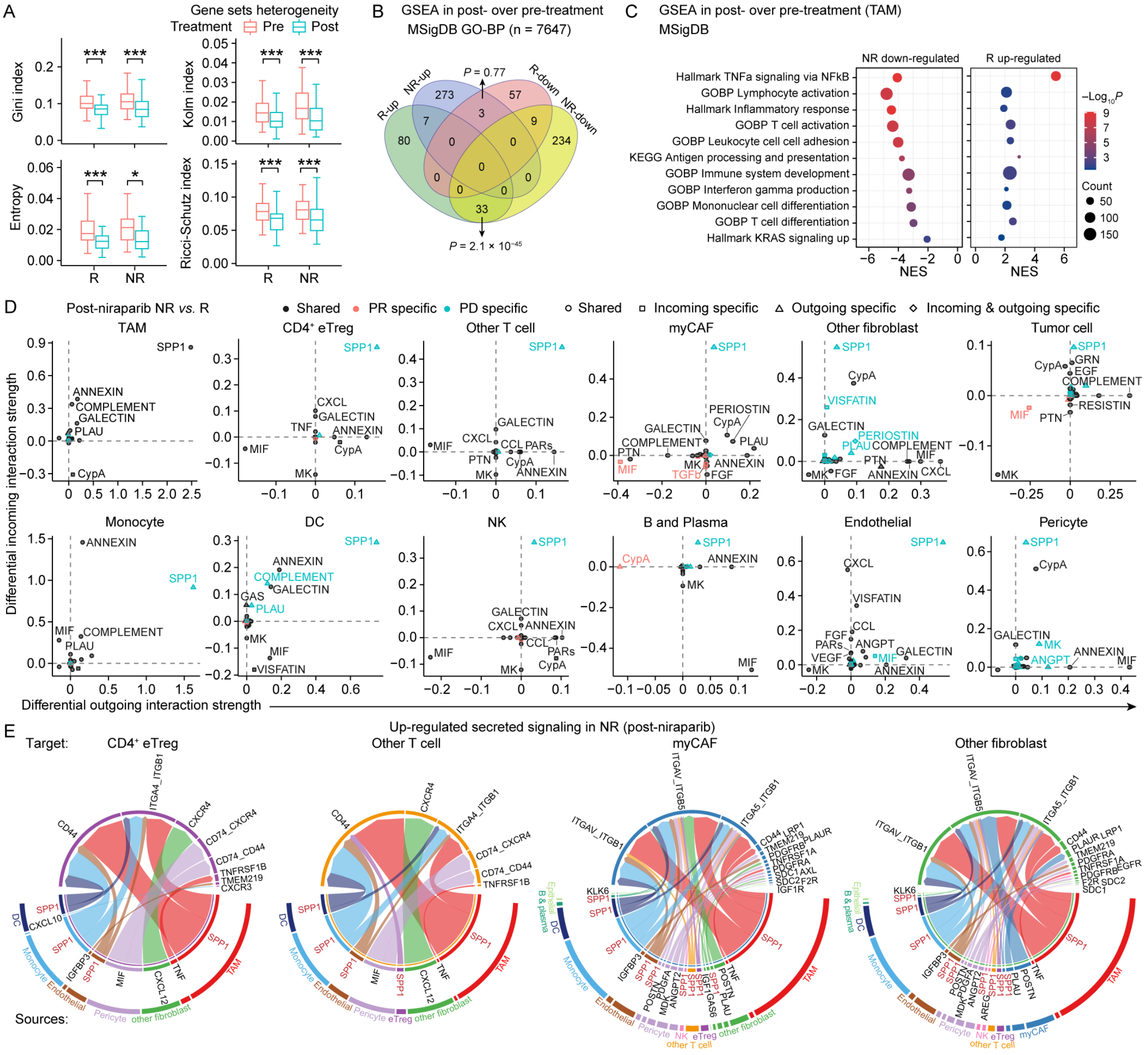

Supplementary Figure 7. SPP1-dominant cellular crosstalk underlies niraparib resistance.

(A) Differences in pathway activity heterogeneity of the TAM subsets between niraparib post-treatment and their paired pre-treatment tumors. The hallmark gene sets (n = 50) from the MSigDB were used. Paired samples t-test. *$P < 0.05$, ***$P < 0.001$. **(B)** Overlap among gene set enrichment analysis (GSEA) of TAMs between niraparib paired pre- and post-treatment tumors in the response (R) group and non-response (NR) group, respectively. Biological Process (BP) gene sets (n = 7647) of the Gene Ontology resource (GO) were used. chi-square test. **(C)** Gene set of TAMs that were simultaneously upregulated in post-niraparib R tumors and downregulated in NR tumors compared to their paired pre-treatment tumors, respectively. **(D)** CellChat comparison of the secreted signaling for the indicated cells between niraparib R and NR groups of post-treatment tumors. **(E)** CellChat delineate up-regulated ligand-receptor pairs of the secreted signaling for the indicated cells in post-treatment NR tumors. For A−E, niraparib R (n = 8) and NR (n = 5) of post-treatment tumors in the scRNA-seq data was used.

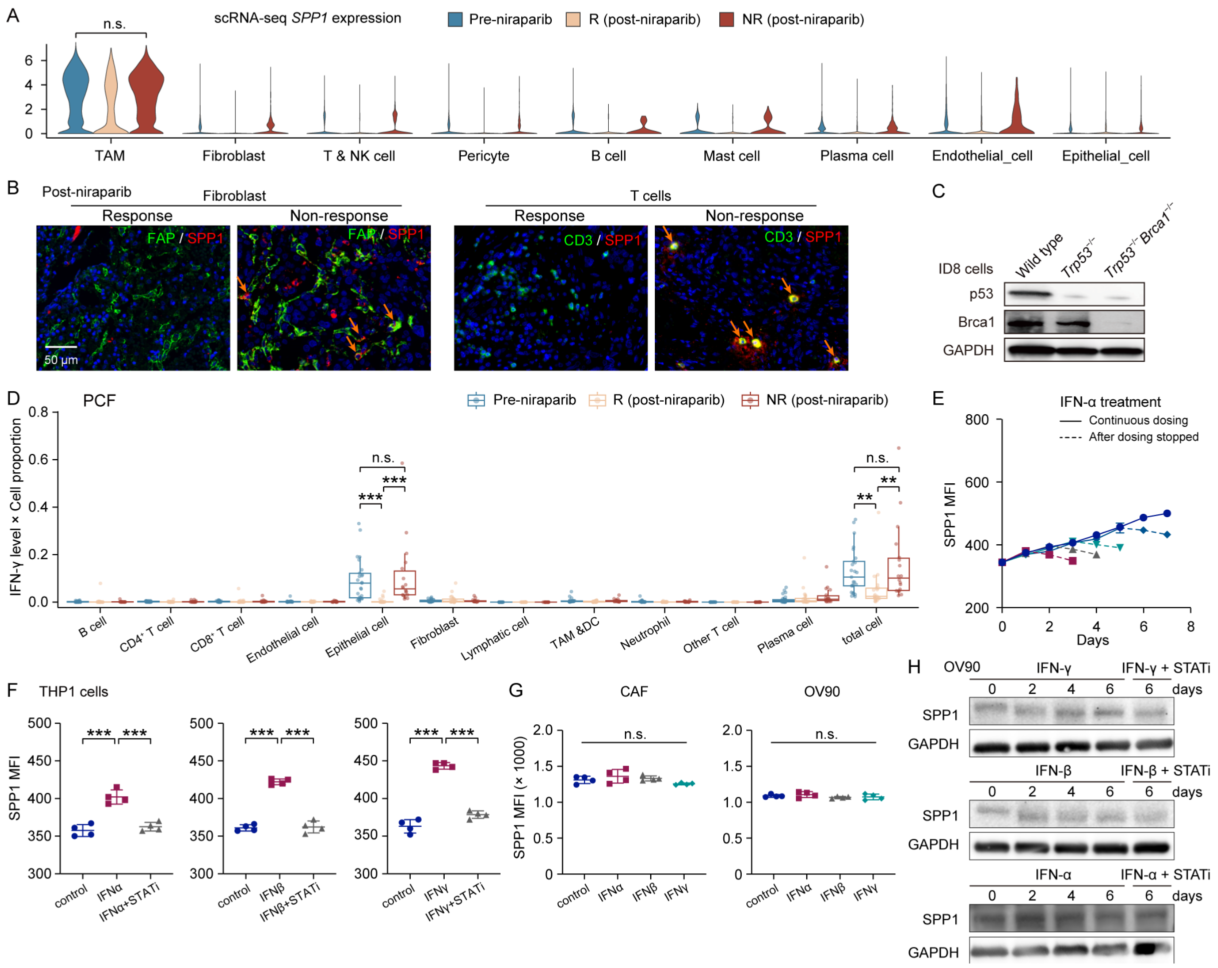

Supplementary Figure 8. IFN induce SPP1 up-regulation in TAM.

(A) Violin plots showing scRNA-seq-derived *SPP1* expression across cell lineages, compared between pre-treatment (n=17), post-niraparib R (n=9) and post-niraparib NR (n=5). **(B)** Representative immunofluorescence images with arrows indicating $FAP^{+}SPP1^{+}$ and $CD3^{+}SPP1^{+}$ cells. **(C)** Western blot validation of Brca1/Trp53 knockout in ID8 ovarian cancer cells. **(D)** Violin plots showing PCF-derived relative IFN-γ expression contribution (defined by normalized IFN-γ × cell proportion) across cell lineages, compared among pre-treatment (n=25), post-niraparib R (n=21) and post-niraparib NR (n=18). Wilcoxon rank-sum test. **(E)** Quantification of SPP1 mean fluorescence intensity (MFI, n=4) following continuous IFN-α treatment; solid line represents continuous IFN-α dosing, while dashed lines indicate SPP1 expression decay after IFN-α withdrawal at specific time points. **(F-G)** Quantification of SPP1 mean fluorescence intensity (MFI, n=4) in THP1 (**F**) and CAF/OV90 (**G**) cells following 3-day treatment with IFN, with or without STAT1 inhibitor (Fludarabine, 2μg/ml). Student's t test. **(H)** Western blot analysis of SPP1 protein levels in OV90 cells following continuous IFNα, IFNβ and IFN-γ treatment (10 ng/mL) with or without STAT1 inhibitor (Fludarabine, 2μg/ml) for the indicated time points. *$P < 0.05$, **$P < 0.01$, ***$P < 0.001$, n.s. not significant.

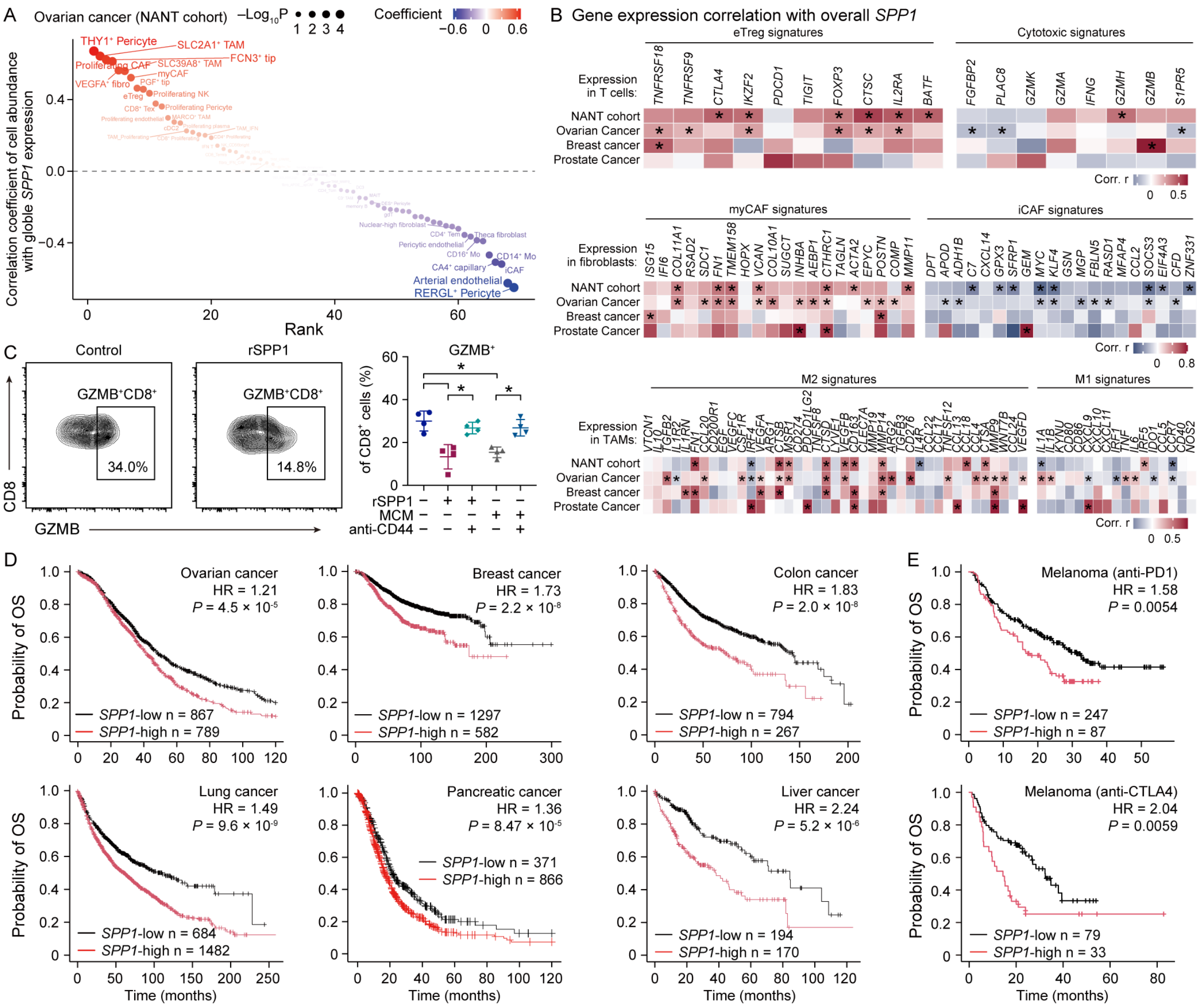

Supplementary Figure 9. SPP1 correlates with immunosuppression and poor prognosis following PARPi therapy.

(A) Rank of the correlation coefficients between global *SPP1* expression and the proportion of each cell subtype relative to their respective major cell types (n = 33). Spearman correlation analysis. **(B)** Correlation analysis between global *SPP1* expression and the expression of marker genes for T cells, myCAF, and TAMs in HGSOC utilizing scRNA-seq datasets of the NANT cohort (n = 33) and external GSE180661 (n = 136), in breast cancer using GSE176078 (n = 26), and in prostate cancer using GSE137829, GSE141445, and GSE157703 (n = 9). Spearman correlation analysis, *P < 0.05. **(C)** Flow cytometry quantification of GZMB in CD8$^+$ T cells with or without macrophage-conditioned medium (MCM), with or without anti-CD44 antibody (n=4). Wilcoxon test with Benjamini-Hochberg correction. *P < 0.05. **(D)** Kaplan-Meier curve of overall survival (OS) plotted for patients across various cancer types, using the KMplot database (www.kmplot.com). Samples were divided into low- and high-expression groups based on the best performing threshold of *SPP1* expression. **(E)** Kaplan-Meier curve of overall survival (OS) was plotted for patients with melanoma treated with anti-PD-1/anti-CTLA-4, utilizing the KMplot database (www.kmplot.com). Samples were divided into low- and high-expression groups based on the best performing threshold of *SPP1*.

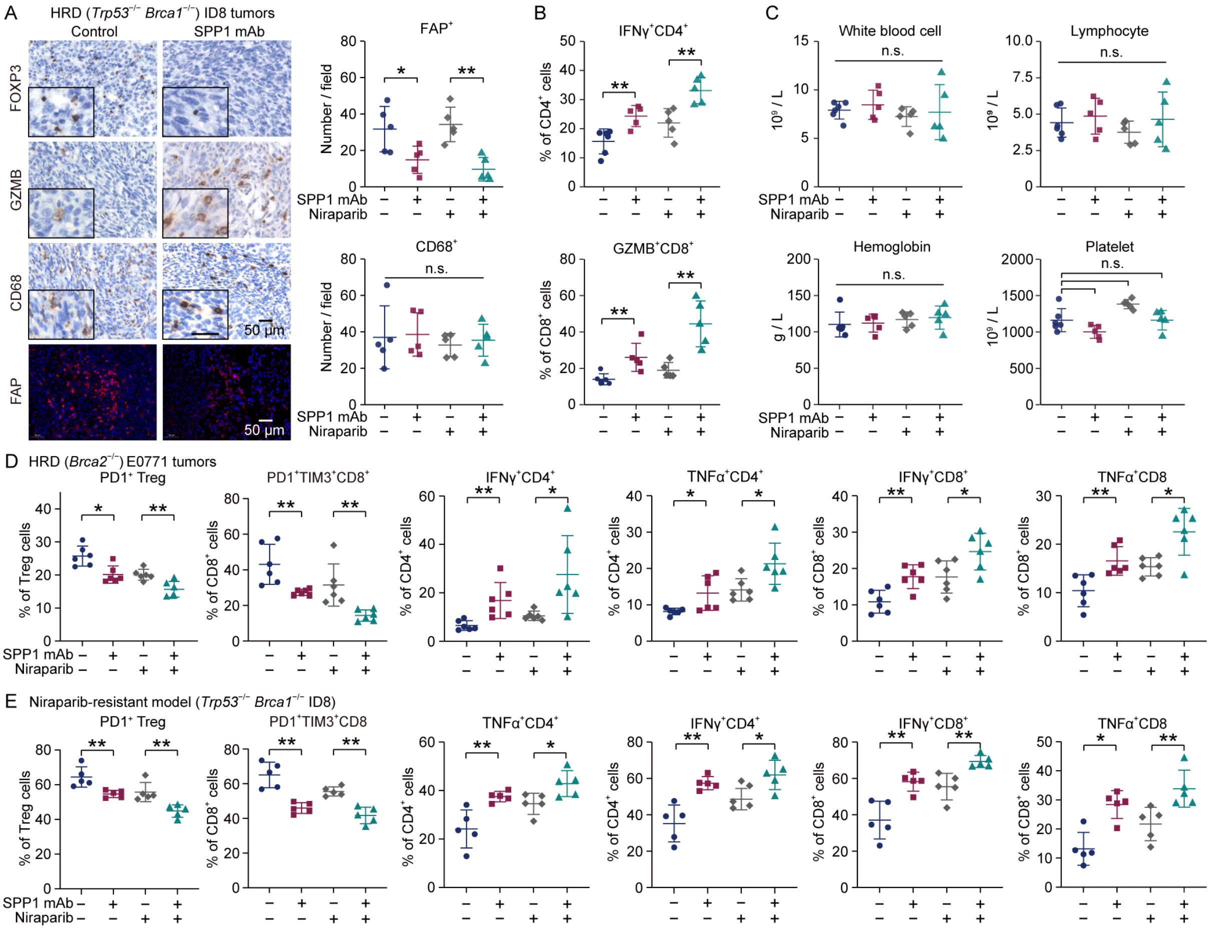

Supplementary Figure 10. SPP1 blockade augments antitumor immunity and overcomes PARP inhibitor resistance.

(A) Representative IHC and immunofluorescent images, with quantification of intratumoral FAP$^+$ and CD68$^+$ cells per field (40× magnification, 5 mice/group). **(B)** Flow cytometric quantification of CD4$^+$IFNγ$^+$ and CD8$^+$GZMB$^+$ T cell subsets in ID8 tumor at endpoint (n = 5 mice/group). **(C)** Hematologic safety profiling of treatment regimens in *Trp53*$^{-/-}$*Brca1*$^{-/-}$ ID8 tumor-bearing mice. Peripheral blood counts (WBC, lymphocytes, hemoglobin, platelets) were analyzed at endpoint (n = 5 mice/group). **(D)** Flow cytometry analysis of indicated cells in E0771 tumors among different groups at endpoint (n = 6 mice/group). **(E)** Flow cytometry analysis of indicated cells in niraparib-resistant ID8 tumors among different groups at endpoint (n = 5 mice/group). Statistical significance was determined by two-tailed Wilcoxon rank-sum test. **P* < 0.05, ***P* < 0.01, n.s. not significant.

REAGENT OR RESOURCES	SOURCE	IDENTIFIER
**Antibodies**		
Anti-human CD163	Abcam	Cat.#ab182422
Anti-human alpha SMA	Cell Signaling Technology	Cat.#19245
Anti-human CD206	Cell Signaling Technology	Cat.#91992
Anti-human CD4	Abcam	Cat.#ab133616 RRID:AB_2750883
Anti-human CD68	Cell Signaling Technology	Cat.#76437
Anti-human CD8 alpha	Abcam	Cat.#ab237709
Anti-human Factor D	Bioss Antibodies	Cat.#bs-13130R
Anti-human fibroblast activation protein alpha	Abcam	Cat.#ab207178
Anti-human FOLR2	Abcam	Cat.#ab302532
Anti-human/mouse FOXP3	Abcam	Cat.#ab215206 RRID:AB_2860568
Anti-human pan-Cytokeratin	Abcam	Cat.#ab7753 RRID:AB_306047
Anti-human PD-1	Cell Signaling Technology	Cat.#86163
Anti-human Periostin	Abcam	Cat.#ab215199
Anti-human TNFRSF9	Cell Signaling Technology	Cat.#19541
Anti-human VFGFA	Abcam	Cat.#ab52917
Anti-human SPP1	Abcam	Cat.#ab214050
Anti-mouse CD68	Cell Signaling Technology	Cat.#97778
Anti-human/mouse Granzyme B	Proteintech	Cat.#13588-1-AP
APC anti-mouse CD4 Antibody	BioLegend	Cat.#100412
PE anti-mouse CD8a Antibody	BioLegend	Cat.#100708
FITC anti-human/mouse Granzyme B Antibody	BioLegend	Cat.#515403
PerCP/Cyanine5.5 anti-mouse IFN-γ Antibody	BioLegend	Cat.#505821
Anti-mouse SPP1	Proteintech	Cat.#229521-1-AP
Anti-mouse fibroblast activation protein alpha	Abcam	Cat.#ab218164
HRP-conjugated goat anti-rabbit IgG antibody	Servicebio	Cat.#G1213
Anti-human/mouse BRCA1	Abcam	Cat.#ab238983
Anti-human/mouse p53	Abcam	Cat.#ab26
Anti-human CD3	Abcam	Cat.#ab16669
Anti-PLC γ1	Immunoway	YM8406
Anti-p-PLC γ1	Immunoway	YM8720
Anti- NF-KB p65	Immunoway	YM8209
Anti- NF-KB p-p65	Immunoway	YM8442
Anti-Akt	Immunoway	YM8463
Anti-p-Akt	Immunoway	YM8304
Anti-S6	Aifang	AFRM0214
Anti-pS6	Cell Signaling Technology	4858S
**Critical commercial assays**		
Chromium Controller	10x Genomics	Cat.#120270
Chromium Next GEM Chip G Single Cell Kit	10x Genomics	Cat.#1000120

Chromium Next GEM Single Cell 3' Gel Bead Kit v3.1	10x Genomics	Cat.#1000122
Chromium Next GEM Single Cell 3' GEM Kit v3.1	10x Genomics	Cat.#1000123
Chromium Next GEM Single Cell 3' Library Kit v3.1	10x Genomics	Cat.#1000157
sCircle^{TM} Single Cell Full Length Immuno_TCR Library Kit Cell	Singleron	Cat.#4153011
Dual Index Kit TT Set A	10x Genomics	Cat.#1000215
Dynabeads Myone Silane	10x Genomics	Cat.#2000048
GEXSCOPE® Single Cell RNA Library Kit Tissue V2	Singleron	Cat.#5180012
KAPA HiFi HotStart ReadyMix PCR Kit	KAPA BioSystems	Cat.#KK2631
**Reagents and Kit**		
Opal 480 Reagent Pack	Akoya Biosciences	Cat.#FP1500001KT
Opal 6-Plex Manual Detection Kit	Akoya Biosciences	Cat.#NEL81100KT
Opal 780 Reagent Pack	Akoya Biosciences	Cat.#FP1501001KT
Red Blood Cell Lysis Solution(10x)	Miltenyi Biotec	Cat.#130-094-183
Dead Cell Removal Kit	Miltenyi Biotec	Cat.#130-090-101
SPRIselect Reagent Kit	Beckman Coulter	Cat.#B23318
Tissue Storage Solution	Miltenyi Biotec	Cat.#130-100-008
Tumor Dissociation Kit, human	Miltenyi Biotec	Cat.#130-095-929
Tumor Dissociation Kit, mouse	Miltenyi Biotec	Cat.#130-096-730
Cell Activation Cocktail	BioLegend	Cat.#423304
Zombie NIR™ Fixable Viability Kit	BioLegend	Cat.#423106
Sextuple-Fluorescence immunohistochemical mouse/rabbit kit	Immunoway	Cat.#RS0039
DAB	Servicebio	Cat.#G1212
**Deposited data**		
Single-cell RNA data	This paper	GSE222557
**Software and algorithms**		
Adobe Illustrator	Adobe	https://www.adobe.com/products/illustrator.html
CeleScope v1.11.0b0	Singleron Biotechnologies	https://github.com/singleron-RD/CeleScope
CellChat v2.1.2	Jin et al. 2021	https://github.com/sqjin/CellChat
clusterProfiler v4.2.2	Yu G, et al. 2012	https://bioconductor.org/packages/release/bioc/html/clusterProfiler.html
DropletUtils v1.10.3	Lun et al., 2019	Bioconductor
edgeR v3.36.0	Robinson MD, et al. 2010	https://bioconductor.org/packages/edgeR
inForm2.6.0	Akoya Biosciences	https://www.akoyabio.com/support/software/
miloR v1.99.9	Dann, E, et al. 2022	https://github.com/MarioniLab/miloR
msigdbr v7.5.1	Subramanian A, et al. 2005	https://www.gsea-msigdb.org/gsea/msigdb
phenoptr v0.3.2	Akoya Biosciences	https://akoyabio.github.io/phenoptr/
Presto v1.0.0	Korsunsky I, et al. 2019	https://github.com/immunogenomics/presto
R v4.2.0	The R Foundation	https://www.r-project.org
SAS 9.4	SAS Institute Inc.	https://www.sas.com/zh_cn/home.html
Seurat v4.1.1	Hao et al., 2021	https://github.com/satijalab/seurat
