# Supplementary material for: Tumor cell–derived IFN spatially reprograms osteopontin-enriched macrophage niches to promote PARP inhibitor resistance
Source: J Clin Invest. 2026 Mar 6;136(8):e199035. doi: 10.1172/JCI199035 (PMC13078868; doi:10.1172/JCI199035)
Supplement: Unedited blot and gel images [file jci-136-199035-s179.pdf]

Figure 5M

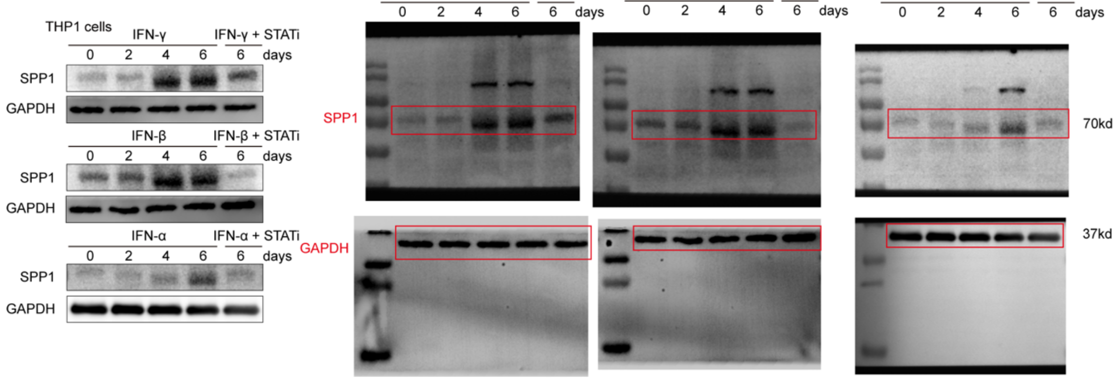

Figure 6C

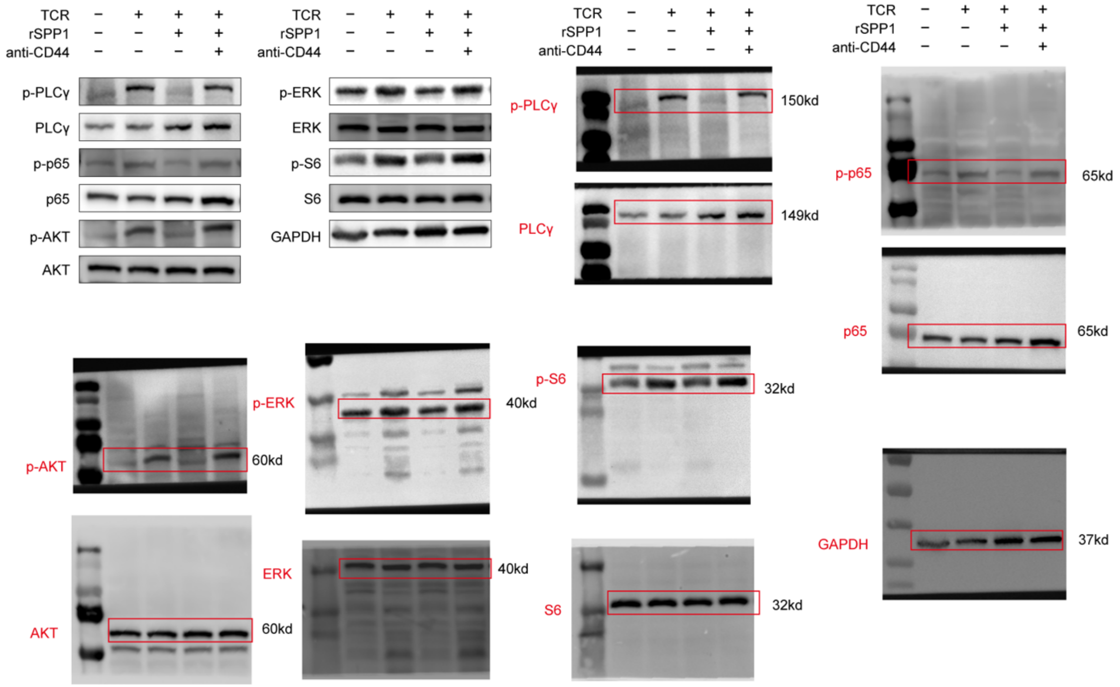

Figure S8C

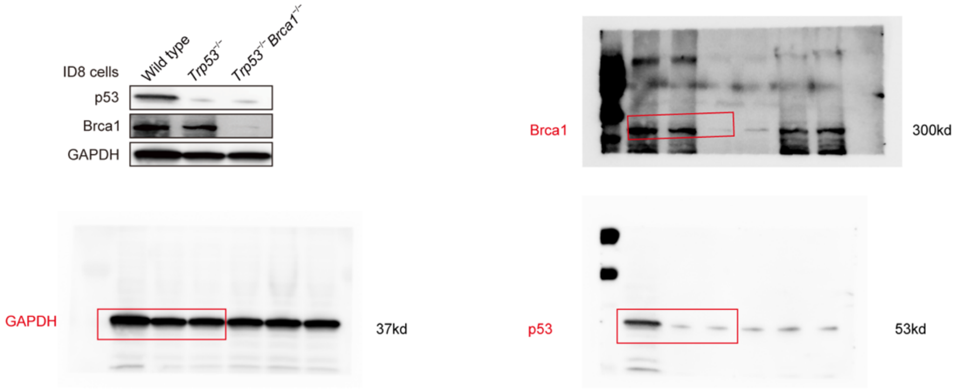

Figure S8H

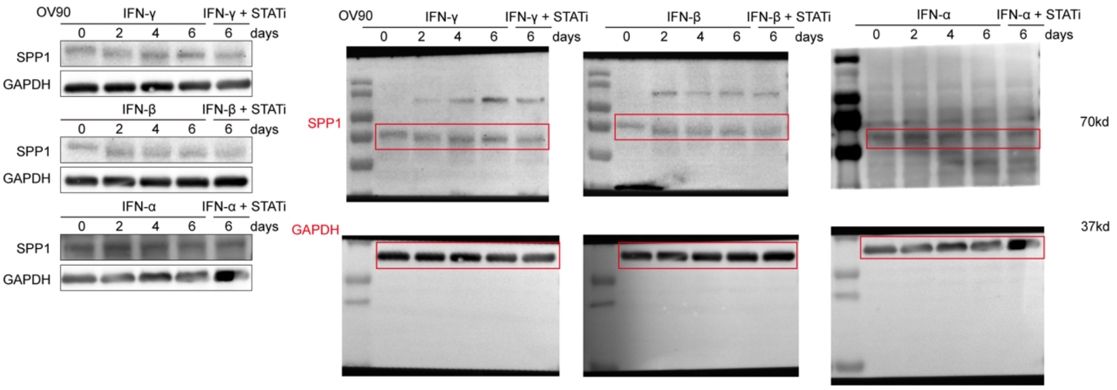
OV90
IFN-γ
IFN-γ + STATi
0 2 4 6 6 days
SPP1
GAPDH
IFN-β
IFN-β + STATi
IFN-α
IFN-α + STATi
70kd
37kd
